# Increased n-6 Polyunsaturated Fatty Acids Indicate Pro- and Anti-Inflammatory Lipid Modifications in Synovial Membranes with Rheumatoid Arthritis

**DOI:** 10.1007/s10753-023-01816-3

**Published:** 2023-05-04

**Authors:** Anne-Mari Mustonen, Sylvain Tollis, Reijo Käkelä, Sanna P. Sihvo, Sanna Palosaari, Vesa-Matti Pohjanen, Aaron Yli-Hallila, Petri Lehenkari, Petteri Nieminen

**Affiliations:** 1 Institute of Biomedicine, School of Medicine, Faculty of Health Sciences, University of Eastern Finland, P.O. Box 1627, FI-70211 Kuopio, Finland; 2 Department of Environmental and Biological Sciences, Faculty of Science, Forestry and Technology, University of Eastern Finland, P.O. Box 111, FI-80101 Joensuu, Finland; 3 Molecular and Integrative Biosciences Research Programme, Faculty of Biological and Environmental Sciences, University of Helsinki, P.O. Box 65, FI-00014 Helsinki, Finland; 4 Helsinki University Lipidomics Unit (HiLIPID), Helsinki Institute of Life Science (HiLIFE) and Biocenter Finland, University of Helsinki, P.O. Box 65, FI-00014 Helsinki, Finland; 5 Cancer and Translational Medicine Research Unit, Faculty of Medicine, University of Oulu, P.O. Box 5000, FI-90014 Oulu, Finland; 6 Medical Research Center, University of Oulu and Oulu University Hospital, P.O. Box 5000, FI-90014 Oulu, Finland; 7 Department of Surgery, Oulu University Hospital, P.O. Box 21, FI-90029 OYS, Oulu, Finland

**Keywords:** fatty acids, inflammation, osteoarthritis, rheumatoid arthritis, synovial membrane, synovium.

## Abstract

Emerging evidence suggests that fatty acids (FAs) and their lipid mediator derivatives can induce both beneficial and detrimental effects on inflammatory processes and joint degradation in osteoarthritis (OA) and autoimmune-driven rheumatoid arthritis (RA). The present study characterized the detailed FA signatures of synovial membranes collected during knee replacement surgery of age- and gender-matched OA and RA patients (*n* = 8/diagnosis). The FA composition of total lipids was determined by gas chromatography and analyzed with univariate and multivariate methods supplemented with hierarchical clustering (HC), random forest (RF)-based classification of FA signatures, and FA metabolism pathway analysis. RA synovium lipids were characterized by reduced proportions of shorter-chain saturated FAs (SFAs) and elevated percentages of longer-chain SFAs and monounsaturated FAs, alkenyl chains, and C20 n-6 polyunsaturated FAs compared to OA synovium lipids. In HC, FAs and FA-derived variables clustered into distinct groups, which preserved the discriminatory power of the individual variables in predicting the RA and OA inflammatory states. In RF classification, SFAs and 20:3n-6 were among the most important FAs distinguishing RA and OA. Pathway analysis suggested that elongation reactions of particular long-chain FAs would have increased relevance in RA. The present study was able to determine the individual FAs, FA groups, and pathways that distinguished the more inflammatory RA from OA. The findings suggest modifications of FA elongation and metabolism of 20:4n-6, glycerophospholipids, sphingolipids, and plasmalogens in the chronically inflamed RA synovium. These FA alterations could have implications in lipid mediator synthesis and potential as novel diagnostic and therapeutic tools.

**Supplementary Information:**

The online version contains supplementary material available at 10.1007/s10753-023-01816-3.

## INTRODUCTION

Synovial membrane forms the soft tissue lining of synovial joints and represents a pivotal anatomical site of pathology in inflammatory joint diseases [1, 2]. The lining layer of healthy synovium is 20–40 μm thick with 2–3 layers of cells, while the sublining layer that contains fibrous connective tissue, blood vessels, and a small number of immune cells is up to 5 mm thick. The lining layer mainly consists of macrophage-like (type A) synoviocytes and fibroblast-like (type B) synoviocytes (FLSs). FLSs produce constituents of synovial fluid (SF) that lubricate the surfaces of articular cartilage. SF also sustains nutrition for chondrocytes, whereas macrophages protect against bacterial infections and clear debris. The fat-% of normal synovium is relatively high, 74% and 91% of it consists of neutral lipids, mostly triacylglycerols (79% of total lipids), and free and esterified cholesterol (8%) [3]. Phosphatidylcholine (PC; 2.3%) is the most abundant phospholipid (PL) followed by phosphatidylserine, -inositol, -ethanolamine (PE), and sphingomyelin (SM). PL biosynthesis by FLSs is known to be stimulated under inflammatory conditions [4]. The most abundant fatty acids (FAs) in normal synovium include 18:1 (39%), 16:0 (21%), 18:0 (11%), 18:2 (7%), and 16:1 (6%) [3].

In inflammatory conditions such as osteoarthritis (OA), synovial membrane undergoes thickening with macrophage and lymphocyte infiltration, hyperplasia, neovascularization, and fibrosis [2, 5, 6]. The increased production of cytokines, nitric oxide, prostaglandin E_2_ (PGE_2_), and cartilage-degrading proteinases by FLSs leads to synovitis, joint swelling, inflammatory pain, and the degradation of the cartilage extracellular matrix. In autoimmune-driven rheumatoid arthritis (RA), the synovial lining layer is converted into a pannus-like structure that attaches to the cartilage surface invading and degrading cartilage and bone [7]. Due to OA and RA, SF undergoes compositional changes with potential influence on its boundary lubrication properties [8]. The inflammatory load (cells and cytokines) is higher in RA SF than in OA SF [9]. Accordingly, the expression of inflammatory genes and the secretion of cytokines are elevated in RA FLSs, which also show a more rapid proliferation rate and a stronger invasion ability [10]. Furthermore, pannus formation increases energy demands (ATP), and the dysregulated microvasculature causes hypoxia [11]. These phenomena can lead to the enhanced production of reactive oxygen species and oxidative stress in RA synovium.

Inflamed joints are characterized by metabolic alterations, and sugar and amino acid metabolism becomes disturbed in RA FLSs [12]. FA oxidation can be down-regulated in RA synovial membrane [13], and recent findings on SF suggest reduced biosynthesis of FAs in RA compared to OA [14]. So far, lipid metabolism profiling has not been the focus of studies on arthritic synovium. However, it would merit exploring whether bioactive lipids could be involved in the pathogeneses of inflammatory joint diseases. Lipids not only play structural roles, but they influence membrane fluidity [15] and the formation of lubricants [16], inflammatory agents [17], and extracellular vesicles [18]. Individual FAs and their derivatives (lipid mediators, LMs) can induce potentially beneficial and detrimental effects on inflammatory processes, joint erosion, and perception of pain [19]. RA SF has been documented to contain higher levels of polyunsaturated FA (PUFA)-derived LMs, including some specialized pro-resolving mediators (SPMs), than OA SF [20]. In synovium, lower signals of PC and SM species were noted in the mass spectrometric images of RA compared to OA patients [21]. To the best of our knowledge, the detailed FA signature of arthritic synovium has not been previously documented in inflammatory joint diseases.

The aim of the present study was to compare (*i*) the FA composition and (*ii*) enzymatic pathways of FA metabolism between synovial membranes of previously diagnosed RA and OA patients. It was hypothesized that (*i*) the proportions of inflammatory FAs would be overrepresented in RA synovium and that (*ii*) these modifications would be reflected in the corresponding metabolic pathways that elongate and unsaturate FAs.

## PATIENTS AND METHODS

### Patients and Sampling

Synovial membrane samples were collected from 8 patients with end-stage OA (2 men and 6 women, average age: 71 ± 3 years, body mass: 76.3 ± 3.88 kg, body mass index [BMI]: 28.3 ± 1.22 kg/m^2^) and 8 patients with end-stage seropositive RA (2 men and 6 women, 69 ± 3 years, 73.3 ± 6.97 kg, 27.2 ± 1.50 kg/m^2^) during knee replacement surgery at the Oulu University Hospital and stored at –80 °C. All RA patients, except one, had a long-standing disease that fulfilled the 1987 diagnostic criteria of the American College of Rheumatology or the 2010 renewed criteria of the American College of Rheumatology/European League Against Rheumatism [22]. Seropositivity was defined by elevated concentrations of either rheumatoid factor or anti-citrullinated protein antibodies, or both. The study was approved by the Ethical Committee of the Hospital (decision #29/2011, amendment 2/24/2014) in compliance with the Helsinki Declaration, and the patients provided written informed consent to donate samples for research. They were surveyed for general data, including gender, age, body mass, height, operative diagnosis, and medication.

Synovium subsamples (*n* = 6/group) were processed into histological sections, stained with hematoxylin and eosin, and semi-quantitatively evaluated for the Krennʼs synovitis score by 3 independent evaluators based on the enlargement of the lining cell layer, cellular density of synovial stroma, and leukocytic infiltrate [23]. Necrosis with a loss of the lining cell layer was observed in 3 RA samples, which affected the synovitis grading by lowering the score of the lining layer hyperplasia. Because of this, the amount of necrosis was also scored to get a more accurate measure of the inflammatory state.

### FA Determination

The subsamples of synovial membranes were transmethylated in methanolic H_2_SO_4_ under N_2_ atmosphere [24]. The formed FA methyl esters (FAMEs) were extracted with hexane and analyzed by the Shimadzu GC-2010 Plus gas chromatograph (Shimadzu, Kyoto, Japan) with the flame ionization detector. Dimethyl acetals (DMAs) produced by the transmethylation of alkenyl chains of plasmalogen PLs were also included in the analysis. The FAME and DMA structures were confirmed by using electron impact mass spectra recorded by the Shimadzu GCMS-QP2010 Ultra with the mass selective detector. The resulting chromatographic peaks were manually integrated with the Shimadzu GCsolution software. The results are expressed as mol-% in total lipid side chains. Related ratios and indices were calculated as described in Mustonen *et al*. [24].

### Statistics and Bioinformatics

#### Statistical Analyses

All statistical analyses were conducted with the IBM SPSS *v*27 software (IBM, Armonk, NY, USA). Comparisons between OA and RA were performed with the Mann–Whitney *U* test and sex ratios were tested with the Fisherʼs exact test. The *p*-value ≤ 0.05 was considered statistically significant. The results are presented as the mean ± SE. We also performed the supervised linear discriminant analysis (DA) by classifying the individual FA data by discriminant functions to observe how clearly OA and RA samples differed from each other, which FAs separated them most clearly, and how accurately the analysis was able to classify the samples into their respective diagnosis. We used diagnosis/tissue type as the grouping variable and individual FAs as the independents. All groups were considered to have equal prior probabilities, and we used the within-groups covariance matrix.

#### Hierarchical Clustering (HC), Correlation Analysis, and FA Group Identification

For each individual FA mol-% value, sum of the FA values in structural categories, ratio, and sample *i*, the measured FA relative abundance ${x}_{i}$ was converted to a *Z*-score using

$${Z}_{i}=\frac{{x}_{i}-{<x>}_{samples}}{{\sigma }_{samples}},$$

where ${<x>}_{samples}$ and ${\sigma }_{samples}$ were, respectively, the mean FA or FA-derived variable across samples and the corresponding standard deviation. This data normalization process was performed within the IBM SPSS software. The data were subsequently loaded onto ClustVis (https://biit.cs.ut.ee/clustvis/) using the variable *Z*-scores as rows and individual samples as columns. HC was performed using “correlations” as the clustering distance and the Ward clustering method with unsquared distances for both rows and columns [25]. Categorical variables (i.e., diagnoses) were not used for clustering. Pearson (linear) correlation analysis was performed in R *v*4.1.2 [26] by using our custom script based on the corrplot library. The clustering of FAs and FA-derived variables (from now on, briefly, “FAs”) into groups was assessed visually from the HC plot, and 5 biologically relevant groups were identified (Supplementary Table S1). The relevance of these groups was further confirmed using the FA–FA correlation matrix, which showed strong intra-group correlations between the profiles of FAs belonging to the same group across samples.

#### Random Forest (RF) Analysis, Data Enrichment, and Feature Importance Quantification

The existence of highly correlated FA groups enabled us to enrich the original dataset using a procedure described below. From the 16 original samples and out of the 44 individual FAs, FA sums, or derived ratios available, only one per group was randomly sampled, and the diagnosis (OA or RA) was copied. This random sampling of the FA groups was repeated 1000 times, yielding an enriched dataset of 1000 × 16 = 16,000 samples (but with only 5 features each, the *Z*-score of the FA that was selected within each group).

Thereafter, the enriched dataset was randomly split into a training dataset (80% of the samples) and a testing dataset (20%). The training dataset was used to parametrize a RF algorithm developed in Python based on the Sklearn Random Forest Classifier package (adapted from https://www.kaggle.com/code/prashant111/random-forest-classifier-tutorial/notebook). Our analysis code was provided as an annotated Jupyter notebook (Supplementary Material S1). The following parameters were used to build the RF classifier: n_estimators = 100, criterion = 'entropy',max_depth = 16,min_samples_split = 2,min_samples_leaf = 1,min_weight_fraction_leaf = 0.0,max_features = 5,max_leaf_nodes = None,min_impurity_decrease = 0.0,bootstrap = True,oob_score = False,n_jobs = -1,random_state = None,verbose = 0,warm_start = False,class_weight = None, ccp_alpha = 0.0,max_samples = None.

To compute the prediction accuracy and the feature importance scores, 100 RFs were built using the procedure above, and the scores were averaged over the 100 RF models. The predictive capacity of the models was tested on the testing dataset. This yielded an excellent prediction accuracy of 99.8%, demonstrating that the subsets of the full dataset generated by sampling each FA group once were equivalent to the full dataset.

The relevance of individual FA groups in discriminating between the inflammatory states (RA or OA) was quantified using the feature_importances function of the Sklearn RF package and estimated from the training datasets only. In the RF classification, a feature gets a high importance score if it appears frequently in the forest at top nodes of the classification trees and reduces the classification uncertainty (the so-called “node impurity”) more than other features [27]. To assess the feature importance for individual FA variables, we also built 100 RF models as described above, but using the full dataset (*Z*-scores of 44 individual FAs/FA sums/derived ratios) without data enrichment. We noted that the prediction accuracy was lower in the absence of FA group-based data enrichment (65%).

### Pathway Analysis

The pathway analysis was conducted with the approach developed by Köhler *et al*. [28], who built the Lipid Network Explorer (LINEX) bioinformatics tool. However, we did not use LINEX in its current version as it did not distinguish positions of the double bonds on the carbon chain for unsaturated FAs. The FA mol-% values were averaged separately for OA patients ${(\langle \mathrm{FA}\rangle }_{OA\ samples})$ and RA patients (${\langle \mathrm{FA}\rangle }_{RA\ samples}$), and a “level change score” was computed from each FA relative to the OA level, chosen as a reference, as

$$\mathrm{Level\ change}(\mathrm{FA})= \frac{{\langle \mathrm{FA}\rangle }_{\mathrm{RA\ samples}}-{\langle \mathrm{FA}\rangle }_{\mathrm{OA\ samples}}}{{\langle \mathrm{FA}\rangle }_{\mathrm{OA\ samples}}}$$

Enzymatic reactions that can convert one FA into another (chain elongation/shortening and desaturation) were obtained from literature [29], and the FA network was manually assembled from these reactions. For each pair of FAs connected by a reaction, the pairwise Pearson correlations between the FA mol-% values were computed within each group of samples separately, and they were classified as significant/nonsignificant depending on their Pearson *p*-value (significance threshold: *p* = 0.05). This was followed by comparing the obtained significances of the FA–FA correlations across the two groups of samples. Strong correlations between 2 FAs, denoted FA1 and FA2, were interpreted as the reaction FA1 → FA2 being the dominant route of FA2 biosynthesis within each diagnosis group, keeping in mind the other possible biosynthetic routes and dietary intake as other sources.

## RESULTS

There were no statistically significant differences in the gender distribution, age, body mass, or BMI of the patients between the diagnoses (Fisherʼs exact test, Mann–Whitney *U* test, *p* = 0.505–1.000). Both RA and OA synovial tissues were characterized by moderate inflammation (grades 4–6) when assessed using the Krennʼs synovitis score. The total scores, scores of the subcategories, and those of necrosis did not differ between the diagnostic groups (Fisherʼs exact test, *p* = 0.182–1.000; Supplementary Fig. S1).

**Fig. 1:**
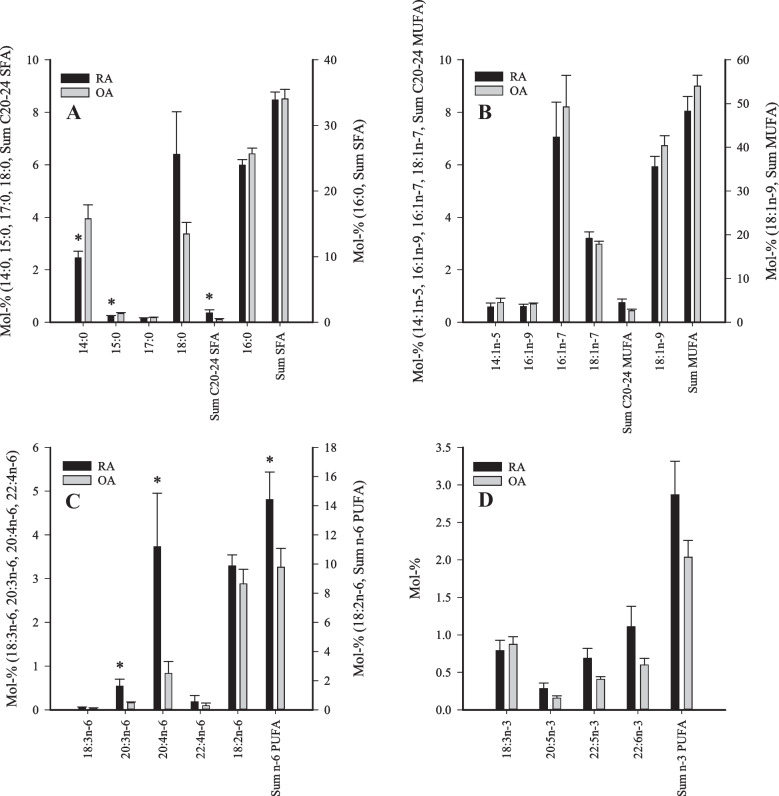
Percentages of selected fatty acids and their sums in arthritic synovium (mol-%, mean + SE). RA, rheumatoid arthritis; OA, osteoarthritis; SFA, saturated fatty acid; MUFA, monounsaturated fatty acid; PUFA, polyunsaturated fatty acid. * = significant difference between the diagnoses (Mann–Whitney *U* test, *p* ≤ 0.05).

Regarding the synovium FA levels, the mol-% values of 14:0 and 15:0 were lower in the RA group than in the OA group, while the percentages of DMA 18:0, 20:3n-6, 20:4n-6, 22:0, 22:1n-9, and 24:1n-9 were higher in the RA patients (Fig. 1, Supplementary Table S2). In addition, total DMAs, total n-6 PUFAs, total PUFAs, product/precursor ratios of n-6 PUFAs, double bond indices, and total average chain lengths were higher in the RA patients compared to the OA patients. The sum of C20–24 saturated FAs (SFAs) was also higher in RA.

**Fig. 2:**
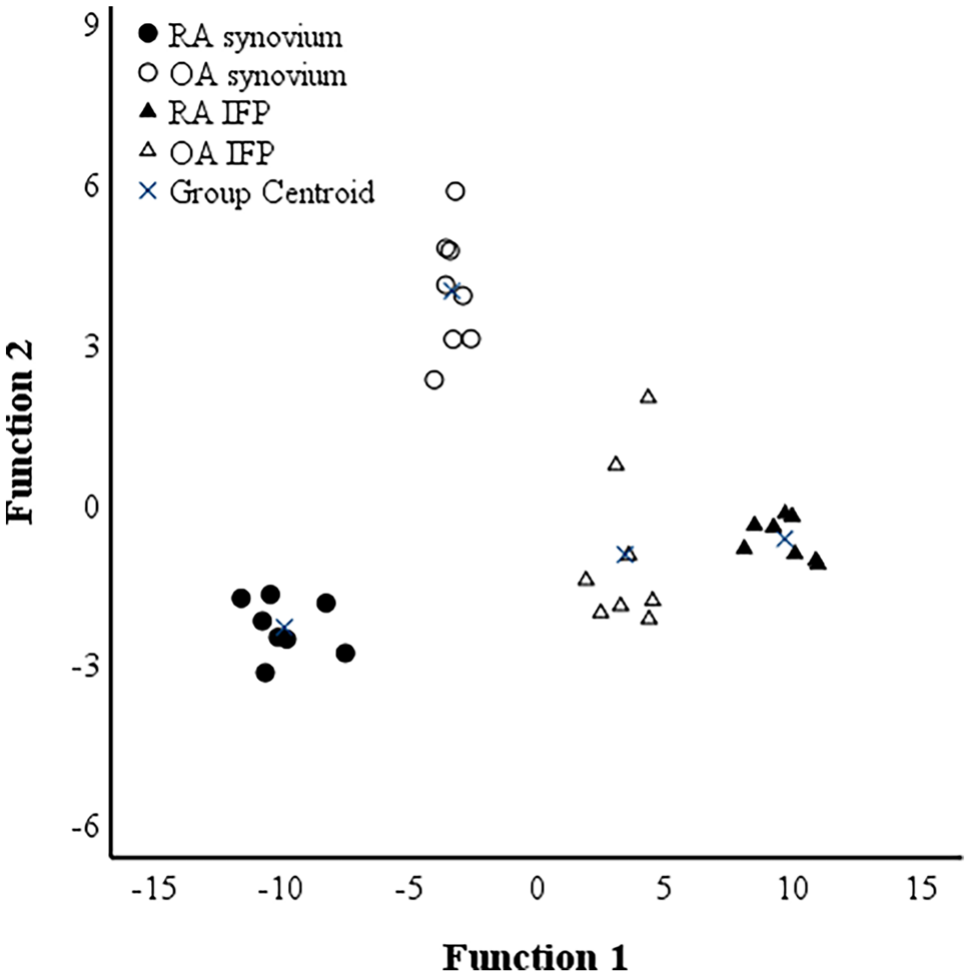
Discriminant analysis depicting the classification of fatty acid data in rheumatoid arthritis (RA) and osteoarthritis (OA) synovial membranes and infrapatellar fat pads (IFPs) based on discriminant functions 1 (on the *x*-axis) and 2 (*y*-axis) that together explain 96.4% of the variance in the dataset. The IFP data derive from Mustonen *et al*. [24].

We next sought to determine whether the differences in the FA composition of synovial membranes could be sufficient to discriminate between RA and OA inflammatory states and, for this purpose, we conducted the supervised DA. Under the assumption that FA composition would be tissue-dependent, we added to the analysis the FA profiles of infrapatellar fat pads (IFPs) from RA and OA patients, which provided a distinct FA composition and served as a positive control for discrimination. These IFP data that were analyzed by the same laboratory with the same equipment and methods have been previously published [24], and 56% of these samples were from the same patients as the synovial membranes. 100% of RA and OA synovium samples were clustered separately from each other and from the RA and OA IFP samples (Fig. 2). Function 1 on the *x*-axis placed synovium samples on the left and IFP samples on the right and clearly separated the RA tissues from each other. Function 2 on the *y*-axis separated the other sample types from OA synovium. The variables having the highest separation power included 22:6n-3, 18:2n-6 (function 1), 14:0, 22:1n-9, 20:0, 20:3n-6, 20:4n-6, and 24:1n-9 (function 2), and functions 1–2 explained 96.4% of variance in the dataset. If IFPs were removed from the DA, function 1 explained 100% of the variance.

**Fig. 3:**
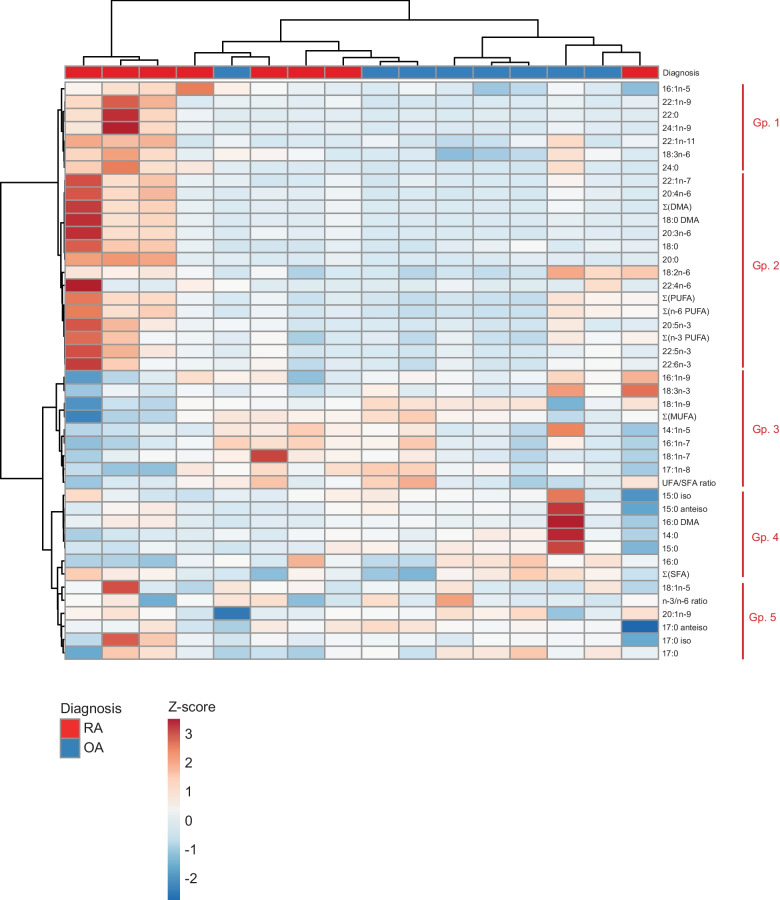
Hierarchical clustering identifies groups of fatty acids (FAs, including dimethyl acetals DMAs), FA sums, and derived ratios (collectively “FAs”) with consistent signatures across diagnoses. Clustergram showing the FA *Z*-scores (rows) in samples (columns) as determined by gas chromatography, color-coded as indicated. For the bonds on the left of the clustergram, the distance of bonds to color-coded clustergram increases with the dissimilarity between FAs across the samples. For the bonds at the top of the clustergram, the distance of bonds to color-coded clustergram increases with the dissimilarity between the samples across the FA space. The clustering was performed with ClustVis using the Ward method [25]. The diagnosis of the corresponding sample (RA, rheumatoid arthritis; OA, osteoarthritis) is indicated on top of the clustergram. Groups (Gp.) of FAs that cluster samples are indicated with red vertical bars on the right side of the image.

**Fig. 4:**
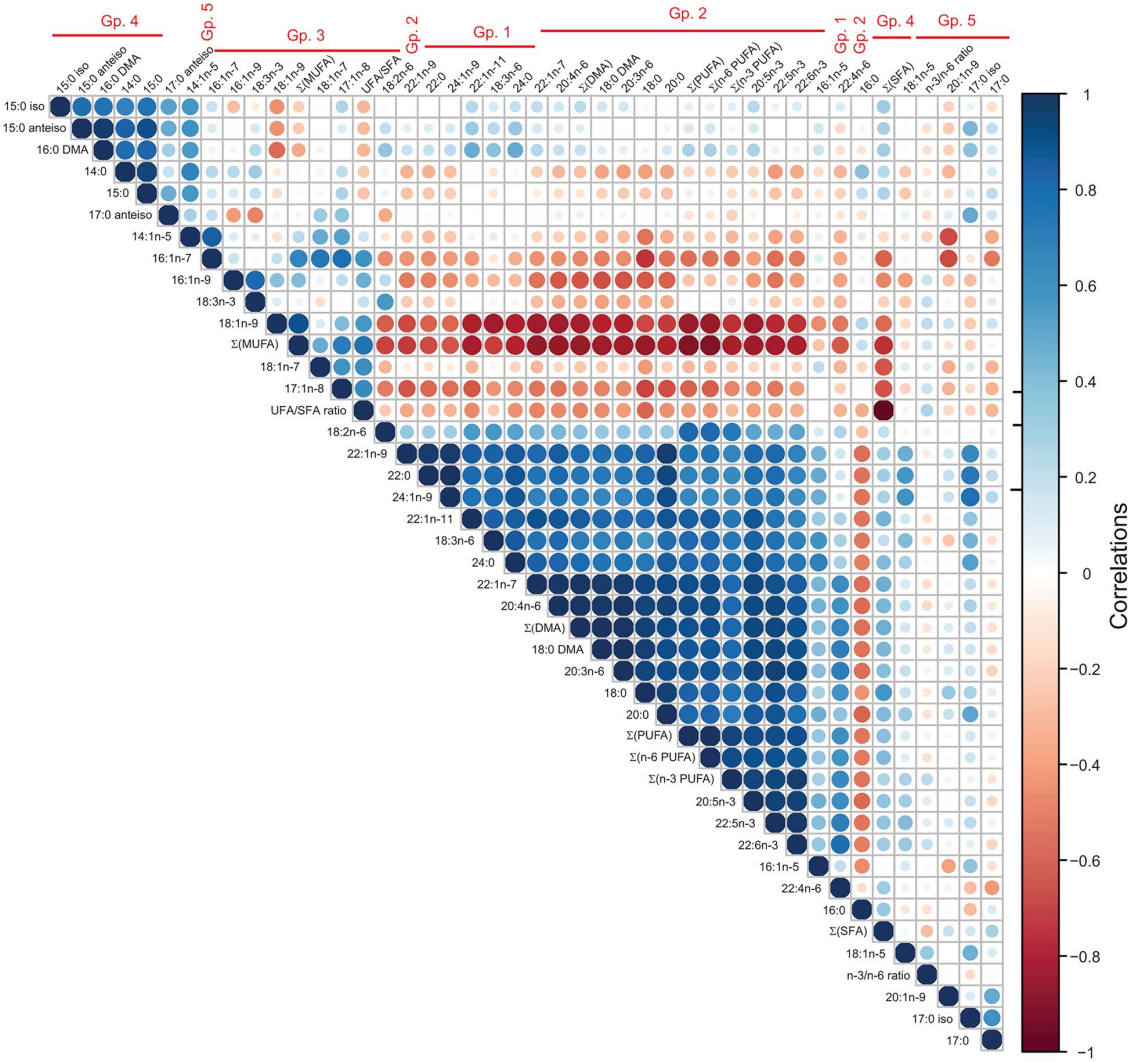
Proportions of fatty acids (FAs), FA sums, and derived ratios (collectively “FAs”) are highly correlated across synovium samples within the FA groups. Correlogram showing the Pearson correlation coefficients between all pairs of FAs across all samples. Dark color (blue or red) indicates strong positive (respectively negative) correlations in the FA levels, irrespective of diagnosis. The correlogram was plotted using the Corrplot function in R. The FA groups (Gp.), as determined by hierarchical clustering across both samples and FAs (Fig. 3), clustered very similarly in the correlogram.

The functions that separated RA and OA samples in the DA tended to include contributions from distinct sets of structurally diverse FAs, indicating that it would be possible to further construct groups of FAs that would show consistent reactions to the studied diagnoses. In order to test this hypothesis, we conducted HC analysis of FA data from RA and OA synovial membranes. HC identified well-defined, biologically meaningful groups of FAs (clusters) with similar patterns across diagnoses, high intra-group similarity, and much lower inter-group similarity (Fig. 3, grouping on the right). Accordingly, intra-group Pearson correlations between any pair of FAs across all samples were typically strong (*r*_p_ > 0.7–0.8), indicating that the intra-group FA compositions mirrored each other across samples while the inter-group correlations were more variable (Fig. 4, correlogram). Based on the HC and correlations, we defined 5 FA groups, within which the variables showed collinearity, indicative of redundant information. Clustering of the diagnoses of the sample donors was only partially achieved (Fig. 3, top rows).

**Fig. 5:**
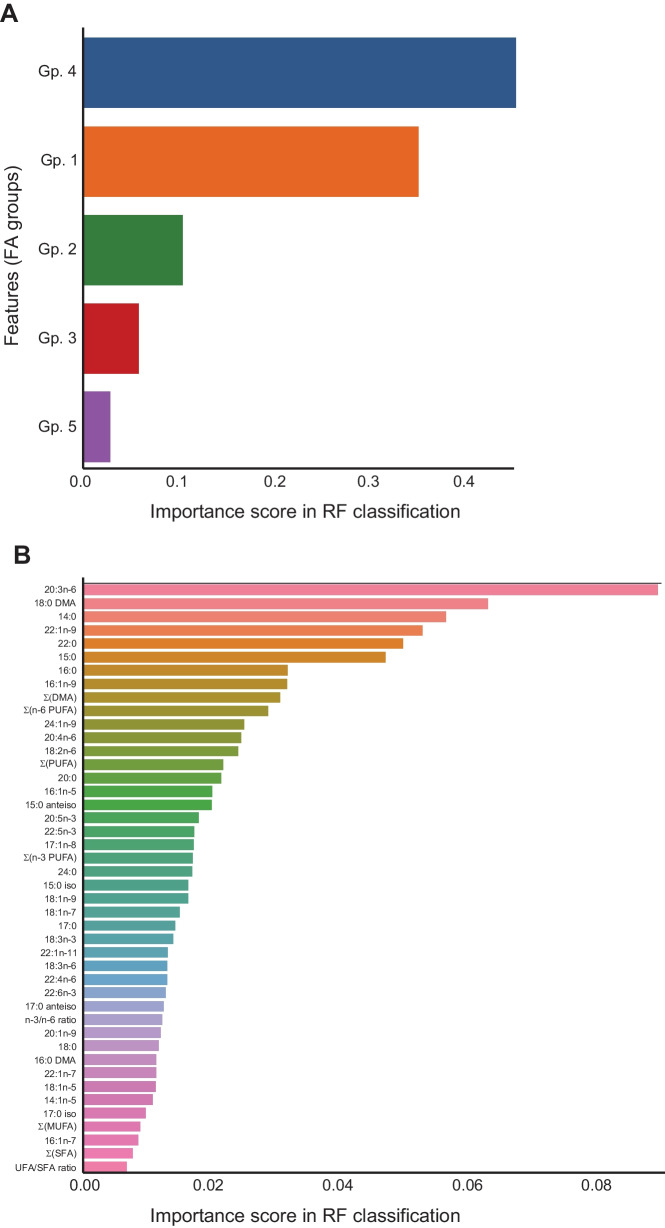
The variables 20:3n-6 and saturated fatty acids (FAs) prevail in random forest (RF)-based classification of the diagnoses of synovium samples. In panel A, bar chart shows the feature importance score of each FA group (Gp.) in the RF-based classification of the diagnosis of the samples (see the “Methods” section). The presented scores are averages of the FA group importance scores over 100 RFs of 100 trees each, run on a 1000-fold enriched dataset. In panel B, bar chart shows the feature importance score for each individual FA, sum, or ratio in the RF-based classification of the diagnosis of the samples. The scores presented are averages of the FA importance scores over 100 RFs of 100 trees each.

In order to test how effectively the identified FA groups performed dimensionality reduction and extracted the functional essence of the FA profiles, we investigated if partial data from each variable group were sufficient to predict the diagnosis of each synovium sample in unsupervised testing (see Methods for details). In this purpose, we performed RF-based classification of the diagnoses, using as classification features the *Z*-scores of only one FA variable per group (randomly selected at each iteration, see the “Methods” section). The RF model constructed on partial data successfully predicted 99.8% of the testing data, demonstrating that providing partial profiles of FAs and their derived variables as training data (sampled within each FA group) was sufficient to predict the health status of all other partial profiles. Hence, there was no loss of inflammatory status-related information upon the reduction of FA signatures to the 5 identified groups. Within these FA groups, group 4, consisting of C14–C16 SFAs, DMA 16:0, and SFA sum, was the most important to the sample classification by the RF model (Fig. 5A). This trend was also present when the RF model was built on the entire dataset, without grouping, where 20:3n-6 and several SFAs were the most important FAs for sample classification, emphasizing their potential importance in the regulation of inflammation (Fig. 5B).

Unlike the accuracy of RF-based classification, the feature importance can in principle be impacted by the presence of linearly correlated features [30, 31]. To control that this was not the case in our dataset, we repeated the exact same analysis procedure but removed the FA structural category sums and derived ratios (e.g., n-6 PUFA sum and n-3/n-6 PUFA ratio) and kept only the raw FA *Z*-scores as features for RF classification (Supplementary Fig. S2). Both with (panel A) and without (panel B) FA grouping, the most important features were very similar to those obtained in the presence of sums and derived ratios (Fig. 5A–B compared with Supplementary Fig. S2A–B).

We next probed the tissue-specificity of the FA groups. In this purpose, we took advantage of our previously published FA profiles of IFPs from RA and OA patients [24] and merged the synovium and IFP FA data into a single dataset that was assessed using the HC and correlation analysis as described above. As > 50% of the IFP samples originated from the same patients as the synovial membranes, we expected this analysis to focus on tissue-specificity, minimizing inter-patient variability. The FA groups that were identified using only synovial membrane samples were recovered to a large extent by either HC (Supplementary Fig. S3) or correlation analysis (Supplementary Fig. S4). Although the overlap was not perfect, it was in agreement with synovium and IFP samples clustering distinctively in the DA (Fig. 2).

**Fig. 6:**
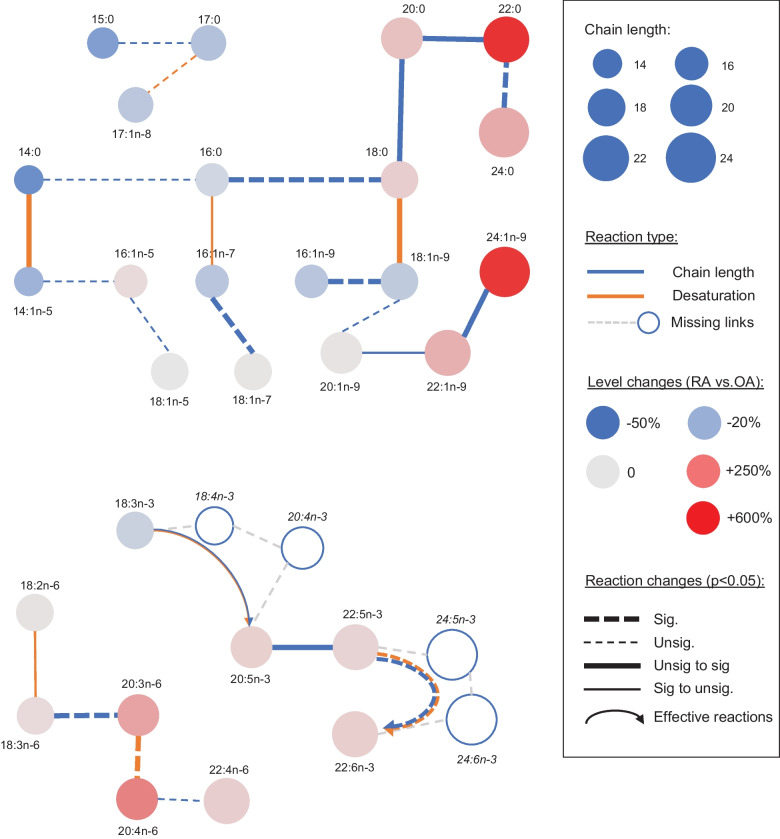
The contribution of elongation to the biosynthesis of long-chain saturated and monounsaturated fatty acids (FAs) increases in rheumatoid arthritis (RA) compared to osteoarthritis (OA). Network view of the FAs included in the dataset (nodes), potentially converted into one another by enzymatic reactions (lines), including alterations in carbon chain length (blue) and desaturation (orange). Node size is proportional to chain length, and node color to the relative change in a FA between OA (reference level) and RA. Changes in the significance of the within-group Pearson correlations between OA and RA FA levels are shown as solid lines (thin: FA–FA correlation is significant across OA samples but not across RA samples; thick: FA–FA correlation is significant across RA samples but not across OA samples). The FA–FA correlations that are either significant or nonsignificant within both groups are shown as thick or thin dotted lines, respectively. Missing nodes and lines in the metabolic reaction chains are indicated as hollow nodes and dashed grey lines, respectively. The FA–FA correlations involving missing parts of the pathway are indicated as curved arrows (following the same nomenclature as other reactions) and referred to as effective reactions.

To get more insight into the physiological basis for the observed differences in the FA composition of synovium in RA *vs.* OA patients, we performed a FA–FA reaction pathway analysis inspired by LINEX [28] (see Methods for details). We discovered that chain elongation reactions of long-chain SFAs and monounsaturated FAs (MUFAs), leading to the enrichment of 22:0 and 24:1n-9, showed increased relevance in RA compared to OA (Fig. 6). The pathway from 18:3n-3 to 20:5n-3 tended to dominate in OA but less so in RA; the opposite was true for the pathway from 20:5n-3 to 22:5n-3, and the one producing 22:6n-3 from 22:5n-3 was as dominant in RA than in OA. Regarding n-6 PUFAs, the pathway leading to 20:4n-6 was dominant in both joint diseases. Most of the reactions involving short-chain FAs were poorly relevant, both in OA and RA.

## DISCUSSION

**Fig. 7:**
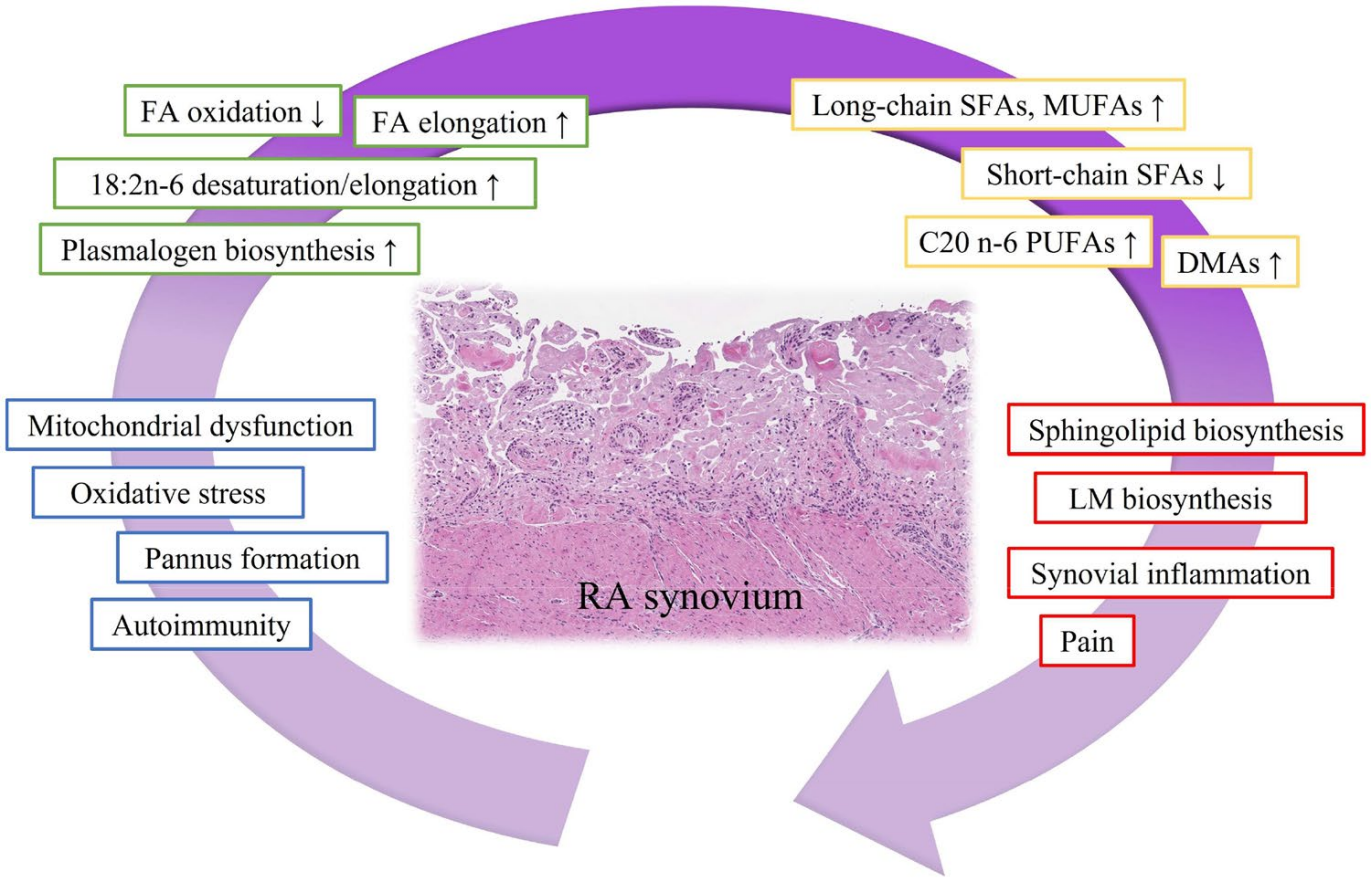
Schematic representation of lipid metabolism alterations associated with rheumatoid arthritis (RA) based on previous literature and present results. FA, fatty acid; SFA, saturated fatty acid; MUFA, monounsaturated fatty acid; PUFA, polyunsaturated fatty acid; DMA, dimethyl acetal; LM, lipid mediator, ↑ = increase, ↓ = decrease.

The present study compared the FA signatures between RA and OA synovial membranes, and with a multi-method approach, we were able to determine the individual FAs, FA groups, and pathways that distinguished the more inflammatory RA from OA. RA was featured with altered proportions of short-chain SFAs (decreased), DMAs, long-chain SFAs and MUFAs, and C20 n-6 PUFAs (increased) compared to OA (Fig. 7). The supervised DA functions computed for the FA profiles of the entire dataset separated 100% of RA synovial membranes from OA membranes. However, a leave-one-out approach of DA revealed that only about 60–65% of the diagnoses could be successfully predicted from the discriminant functions derived from all other samples (data not shown). This indicates that without prior assignment into respective diagnostic groups, the predictive power of the DA remains inadequate to diagnose joint diseases based solely on FA profiles. In the unsupervised HC using the dataset of 16 synovial membranes, FA profiles were also insufficient to wholly predict the RA and OA diagnoses. It is worth remembering that the principal goal of the project was to assess which FAs and FA groupings would be the most significant regarding OA and RA pathogeneses and not to develop a novel diagnostic method. In the RF models, SFAs and 20:3n-6 emerged as the most important FAs for distinction between RA and OA. In addition, the pathway leading to 20:3n-6 and 20:4n-6 from 18:3n-6 was observed to be a potentially significant contributor to both inflammatory joint diseases.

RA was characterized by lower proportions of 14:0 and 15:0 in synovial membranes compared to OA. The potential roles these short-chain SFAs could play in arthritis remain insufficiently understood. In the inflamed RA joint, increased energy demands due to pannus formation [11] could lead to elevated mitochondrial *β*-oxidation of 14:0, which is a preferred substrate compared to longer SFAs. Additionally, ∆9-desaturation and elongation may contribute to its loss in RA synovium [32]. SF 14:0 was previously suggested as a putative biomarker with lower levels in RA than in non-RA inflammatory arthritis, such as ankylosing spondylitis, Behçet’s disease, and gout [33]. In another study, 14:0 levels were higher in RA FLSs compared to OA FLSs [12]. According to Frommer *et al*. [34], 14:0 is able to increase interleukin-6 secretion from RA synovial fibroblasts and normal chondrocytes. In contrast to longer SFAs, 16:0 and 18:0, which can promote cartilage destruction and subchondral bone changes, 14:0 may have protective influence on joints, at least in a rodent model of OA [35]. 15:0 is a minor odd-chain SFA, the plasma levels of which can be associated with the consumption of dairy fat [36]. In addition to biosynthesis by rumen microbiome, 15:0 may be produced by the microbiome of human gut, endogenously de novo (using an odd-chain primer instead of acetyl-CoA in FA synthesis), or by peroxisomal *α*-oxidation [37]. In contrast to even-chain SFAs, 15:0 shows an inverse relationship with coronary heart disease and type 2 diabetes risks. Its levels in serum and SF were inversely correlated with OA severity in a rodent model [38].

The present study documented higher DMA 18:0 and total DMA levels in RA compared to OA synovium. These molecules are plasmalogen derivatives produced from alkenyl chains of plasmalogen PLs during transmethylation. Plasmalogens have attracted increased research interest as they act as antioxidants and protect cells against reactive oxygen species [39] and, recently, they have been associated with several pathological conditions, such as Alzheimerʼs disease, cancer, and cardiovascular disease [40]. Production of PE plasmalogens by FLSs can be elevated under inflammatory conditions [4]. Kosinska *et al*. [41] documented increased levels of 20:4n-6- and 22:6n-3-containing PE plasmalogens in RA and OA SFs compared to control SF and, in a mouse OA model, plasmalogen derivatives in SF showed significant associations with OA severity [38]. According to Koh *et al*. [42], ether-linked PCs increased with inflammation in RA SF. Regarding synovial membranes, psoriatic arthritis was characterized by higher PE plasmalogen levels than OA [21]. Increased DMA levels could be hypothesized to reflect a compensatory mechanism by which tissues counteract the enhanced production of reactive oxygen species and oxidative stress in RA [11] (Fig. 7).

Particular long-chain MUFAs and the sum of long-chain SFAs increased in RA compared to OA synovial membranes, and the pathway analysis suggested that these FAs were increasingly produced by enzymatic reactions in the inflammatory condition of RA. In agreement, previous studies have documented higher levels of C20–22 SFAs in RA FLSs than in OA FLSs [12] and elevated C20–24 MUFAs in RA SF compared to OA SF [43]. The homeostasis of long-chain FAs is known to be important for maintaining normal tissue and cellular functions, and mutations in genes of their synthesis and degradation can cause several inherited disorders [44]. Long-chain SFAs and MUFAs are constituents of sphingolipids [44] that have been associated with inflammation-linked diseases [45] and show elevated levels in RA and OA SFs [41]. It is also known that *β*-oxidation of FAs can be reduced in RA synovial membranes compared to controls [13] and that RA is characterized by mitochondrial dysfunction [11]. It can be hypothesized that besides increased elongation, insufficient oxidation of long-chain FAs could cause their accumulation in RA synovial membrane (Fig. 7). The third hypothesis brings forth the elevated energy requirements of proliferating RA FLSs compared to more quiescent OA FLSs [12], which may result in higher relative utilization of shorter-chain FAs [46]. The observed poor relevance of reaction chains involving some short-chain FAs in both RA and OA suggests that these FAs are largely derived from the diet and their endogenous production is low.

Decreased n-3 PUFA levels were previously documented in RA plasma and SF [43], but this finding could not be reproduced for the RA synovial membranes of the present study. In fact, the pathways leading to the synthesis of particular long-chain n-3 PUFAs appeared as dominant biosynthetic routes in both joint diseases. Regarding n-6 PUFAs, we documented elevated proportions of 20:3n-6, 20:4n-6, and total n-6 PUFAs and stable n-3/n-6 PUFA ratios in RA. Indications of increased desaturation and elongation of 18:2n-6 to C20 n-6 PUFAs were also previously reported regarding the serum PLs of RA patients [47, 48] (Fig. 7). Hypothetically, the increased PUFA percentages in RA samples could reflect a higher load of inflammatory cells (and their membrane PLs) in RA joints [9, 49]. Higher levels of inflammation could also stimulate PL biosynthesis by FLSs [4]. Based on previous literature [19], the effects of OA and RA on the n-6 PUFA levels of synovial joints have been variable, and n-6 PUFAs may induce a combination of beneficial and detrimental effects on joint tissues through their LMs. While 20:3n-6 can be converted into 20:4n-6 by ∆5-desaturase, it is also the precursor for 1-series prostaglandins with anti-inflammatory properties [50]. LMs have the potential to influence the “tumor-like” aggressive phenotype of FLSs that is a hallmark of RA [19]. For instance, PGE_1_ was reported to reduce the proliferation of adherent synovial cells [51] and to decrease the release of matrix metalloproteinase-1 by FLSs [52]. Interestingly, bioinformatic analyses highlighted 20:3n-6 and several SFAs as relevant molecules also in severe equine asthma, emphasizing their potential importance as intermediates or effectors in chronic inflammation [53], and 20:3n-6 was previously linked to diverse inflammatory conditions [54–56].

RA synovium can have higher levels of secretory phospholipase A_2_ (PLA_2_) than OA synovium [57]. PLA_2_ releases 20:4n-6 from cell membrane PLs to LM synthesis and is associated with inflammation. 20:4n-6 and its derivative PGE_2_ can induce the proliferation of synovial cells, leading to pannus formation, while the precursor, 20:3n-6, shows opposite effects [51, 58]. In addition to adverse influence, 20:4n-6 and PGE_2_ have been documented to have beneficial effects on joints [19]. According to Panasyuk *et al*. [59], 20:4n-6 derivatives could inhibit DNA synthesis in chondrocytes and synovial fibroblasts and stimulate proteoglycan and collagen synthesis in chondrocytes. Generally, 20:4n-6 inhibits bone resorption, and PGE_2_ promotes bone formation and enhances resorption [60, 61]. The numerous derivatives of 20:4n-6 include, for instance, 15-deoxy-∆^12,14^-prostaglandin J_2_ (15d-PGJ_2_) and lipoxin A_4_ (LXA_4_) that can induce beneficial effects on joint tissues. 15d-PGJ_2_ is able to trigger synoviocyte apoptosis and to inhibit pannus formation [62], while LXA_4_ suppresses the synthesis of inflammatory and matrix-degrading factors by synovial fibroblasts [63]. PGE_2_ can also inhibit the overgrowth of synovial tissue [64], which makes the connection of n-6 PUFAs to pannus formation even more complex.

The HC-based FA grouping followed by the RF analysis demonstrated that reducing the individual FAs into 5 groups did not cause a loss of information, indicating that the relevant lipid status of arthritic patients could be determined without the analysis of dozens of individual FAs. One such approach would be to determine the concentrations of a few individual indicator FAs of RA and not their relative abundances. Alternatively, a specific FA ratio comparing some indicator FAs that increase with RA against a few decreasing or stable denominator FAs would also allow a quick and sensitive screening of numerous clinical samples. We also included another tissue, IFP, to assess if the separation of the diagnostic groups could be further improved by widening the pool of tissue material. The FA groups identified by using only synovial membrane samples were recovered to a large extent by either HC or correlation analysis with both synovium and IFP samples. As a conclusion, it can be stated that the addition of IFP would not yield further gains in the reduction of numerous FAs into simpler groups and that the synovium and IFP have different general FA profiles, as also evidenced by the DA.

In addition to RA, autoimmune diseases (ADs) in general have been reported to feature a distinct metabolic fingerprint, and several of the FAs noted to be altered in the RA synovium of the present study have been previously associated with different ADs [65]. In contrast, n-3 PUFAs that are precursors for SPM biosynthesis by synoviocytes [66] and that have been suggested to lower the risk for ADs [67] did not differ in proportion between RA and OA synovial membranes in the present study material. It is possible that FA signatures reflect the autoimmune state of the body and could be used as diagnostic clues or therapeutic targets. In fact, evidence has emerged to suggest that ADs may be suppressed by LMs and, thus, there is future potential for LXA_4_, resolvins, protectins, maresins, and their PUFA precursors in the prevention and treatment of ADs, while remembering that 20:4n-6 is also a precursor for pro-inflammatory molecules [68]. This complex web of simultaneously beneficial and detrimental effects of 20:4n-6 metabolism further emphasizes the need for a multi-layered approach to the issue of lipids and inflammation, including lipidomics, bioinformatics, and pathway analyses.

Potential limitations of the present study include the relatively small number of samples and the unfortunate but common difficulty of obtaining a healthy control group. It was somewhat unexpected that the Krennʼs synovitis scores would not differ between RA and OA patients, but this could have been due to the intensive but clinically necessary medication of the RA group (Supplementary Table S3). While this probably also dampened possible differences in the FA profiles between groups, it would have been unethical to withdraw treatment from RA patients based on research requirements, as the knee joint is not the only anatomical location where RA targets synovial tissue. The diagnoses of rheumatic and non-rheumatic joint diseases are not always straightforward, and OA can be a complication of RA or sometimes coexist with it. Here, diagnostics were conducted by experienced rheumatology specialists based on established criteria of clinical findings and seropositivity. Lipid profiling would obviously not be a practical strategy to differentiate between RA and OA but, in the same manner, open biopsies of synovial membrane, while potentially revealing, are invasive and not a realistic form of early diagnostics. They could be conducted during joint replacement surgery, but the diagnoses of both RA and OA practically always precede operative therapy over several years or decades. Instead, FA profiling can provide new data about metabolic differences between patients with established RA and OA, as RA is a more systemic disease, while OA is more localized in particular joints. Thus, the tissue FA signatures of RA patients would also reflect the underlying autoimmune reaction, regardless of the local condition of a particular biopsy.

## CONCLUSIONS

Accumulation of long-chain SFAs and MUFAs, DMAs, and C20 n-6 PUFAs was observed in RA compared to OA synovial membranes. In addition to several SFAs, the proportionally minor n-6 PUFA 20:3n-6 emerged as a potential and promising biomarker for RA inflammation in statistical and bioinformatic analyses, and its role in various inflammatory conditions warrants active further research. The observed FA modifications could be associated with reduced FA oxidation, increased elongation of long-chain SFAs and MUFAs, elevated sphingolipid biosynthesis, and increased abundance of plasmalogen PLs in autoimmune-driven RA (Fig. 7). The alterations in FA metabolism may be involved in the pathogenesis of RA but also counteract the inflammatory processes and oxidative stress in synovial joints. The potential of RA-specific FA signatures as novel diagnostic and therapeutic tools warrants further studies to be elucidated.

## Supplementary Information

Below is the link to the electronic supplementary material.

Supplementary Figure S1. The Krennʼs synovitis scores (mean + SE) in the synovial tissues of osteoarthritis (OA) and rheumatoid arthritis (RA) patients (n = 6/group) (panel A) and representative images of hematoxylin–eosin-stained histological sections of OA and RA synovial tissues (panels B–C), scalebar 100 μm. There were no significant differences in the synovitis scores between the diagnoses. In panel B, OA synovial tissue shows slight hyperplasia, moderate stromal activation with increased vasculature, and moderate inflammatory infiltration. In panel C, RA synovial tissue shows superficial necrosis of the lining layer and thickening of the synovial stroma (PDF 664 KB)

Supplementary Figure S2. The variables 20:3n-6 and saturated fatty acids (FAs) prevail in Random Forest (RF)-based classification of synovium samples based on individual FAs only, excluding FA sums and derived ratios. In panel A, the bar chart shows the feature importance score of each FA group (Gp.) in the RF-based classification of the diagnosis of the samples (see Methods). FA groups were obtained from the groups shown in Fig. 3, where sums and derived ratios were removed. The presented scores are averages of the FA group importance scores over 100 RFs of 100 trees each, run on a 1000-fold enriched dataset. In panel B, the bar chart shows the feature importance score for each individual FA in the RF-based classification of the diagnosis of the samples. The scores presented are averages of the FA importance scores over 100 RFs of 100 trees each (PDF 136 KB)

Supplementary Figure S3. Fatty acid** (**FA) groups are also identifiable across tissues and bear minimal tissue-dependence. Hierarchical clustering clustergram showing the FA Z-scores (rows) in samples (columns) as determined by gas chromatography, color-coded as indicated. For the bonds on the left of the clustergram, the distance of bonds to color-coded clustergram increases with the dissimilarity between FAs across the samples. For the bonds at the top of the clustergram, the distance of bonds to color-coded clustergram increases with the dissimilarity between the samples across the FA space. The clustering was performed with ClustVis using the Ward method [25]. The diagnosis and the tissue of origin of the corresponding sample (RA = rheumatoid arthritis; OA = osteoarthritis; IFP = infrapatellar fat pad) are indicated on top of the clustergram. FA groups (Gp.), identified in Fig. 3, are indicated with red vertical bars on the right side of the image (PDF 220 KB)

Supplementary Figure S4. Fatty acid (FA) proportions are well correlated across diagnoses and tissues within FA groups. Correlogram showing the Pearson correlation coefficients between all pairs of FAs, FA sums, and derived ratios across all samples (RA and OA, synovial membranes and infrapatellar fat pads). Dark color (blue or red) indicates strong positive (respectively negative) correlations in the FA levels, irrespective of diagnosis. The correlogram was plotted using the Corrplot function in R. The FA groups (Gp.), identified in Fig. 3, are indicated on the top of the correlogram. (PDF 409 KB)

Supplementary Material S1. Random Forest analysis code in Python as an annotated Jupyter notebook (Mustonen_et_al_RF_analysis.ipynb) together with the fatty acid level Z-scores for synovial membranes (Synovial_withZscores.xlsx) provided as a single.zip archive (Mustonen_et_al_RF_analysis.zip). (ZIP 228 KB)

Supplementary Table S1. Grouping of fatty acids, dimethyl acetals (DMAs, derivatives of plasmalogen phospholipid alkenyl chains), their sums, and derived ratios based on hierarchical clustering (PDF 411 KB)

Supplementary Table S2. Proportions (mol-%) of fatty acids and alkenyl chains (detected as dimethyl acetal derivatives DMAs) and their sums and ratios in the synovium of rheumatoid arthritis (RA) and osteoarthritis (OA) patients (mean ± SE) (PDF 314 KB)

Supplementary Table S3. Medical data of the patients with rheumatoid arthritis (PDF 180 KB)

## Data Availability

All relevant data analyzed during this study are included in this published article and its supplementary information files.

## Abbreviations

AD: Autoimmune disease
BMI: Body mass index
DA: Discriminant analysis
DMA: Dimethyl acetal
15d-PGJ_2_: 15-Deoxy-∆^12,14^-prostaglandin J_2_
FA: Fatty acid
FAME: Fatty acid methyl ester
FLS: Fibroblast-like synoviocyte
HC: Hierarchical clustering
IFP: Infrapatellar fat pad
LINEX: Lipid Network Explorer
LM: Lipid mediator
LXA_4_: Lipoxin A_4_
MUFA: Monounsaturated fatty acid
OA: Osteoarthritis
PC: Phosphatidylcholine
PE: Phosphatidylethanolamine
PGE_1/2_: Prostaglandin E_1/2_
PL: Phospholipid
PLA_2_: Phospholipase A_2_
PUFA: Polyunsaturated fatty acid
RA: Rheumatoid arthritis
RF: Random forest
SF: Synovial fluid
SFA: Saturated fatty acid
SM: Sphingomyelin
SPM: Specialized pro-resolving mediator
