# Supplementary figures and images for: Increased n-6 Polyunsaturated Fatty Acids Indicate Pro- and Anti-Inflammatory Lipid Modifications in Synovial Membranes with Rheumatoid Arthritis
Source: Inflammation. 2023 May 4;46(4):1396–413. doi: 10.1007/s10753-023-01816-3 (PMC10359413; doi:10.1007/s10753-023-01816-3)

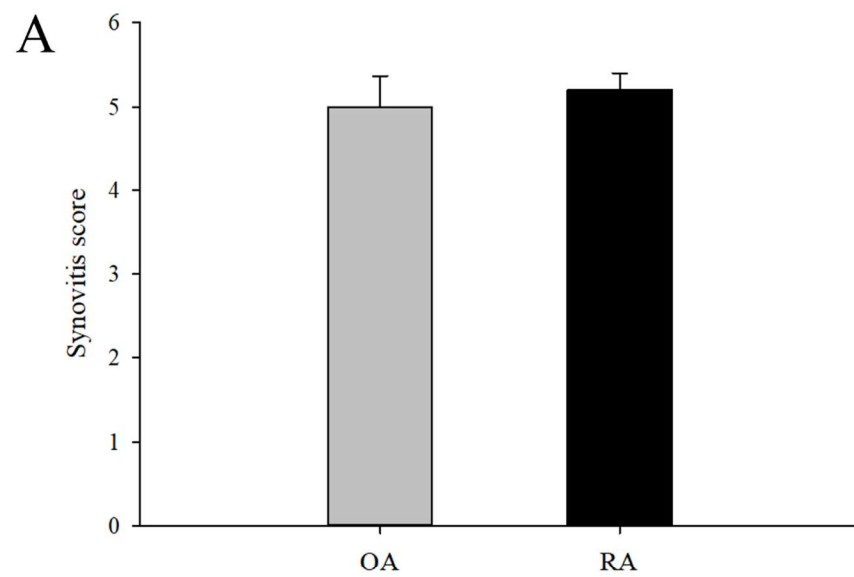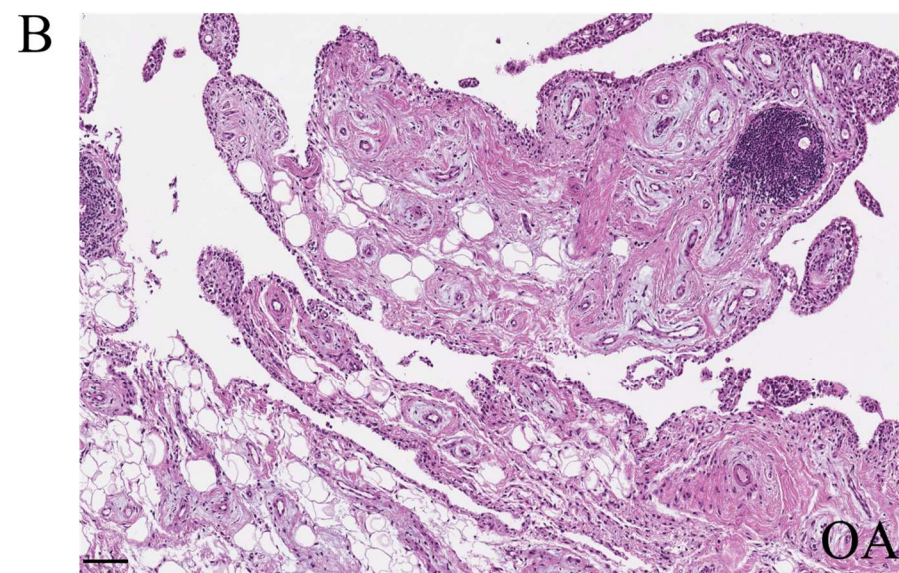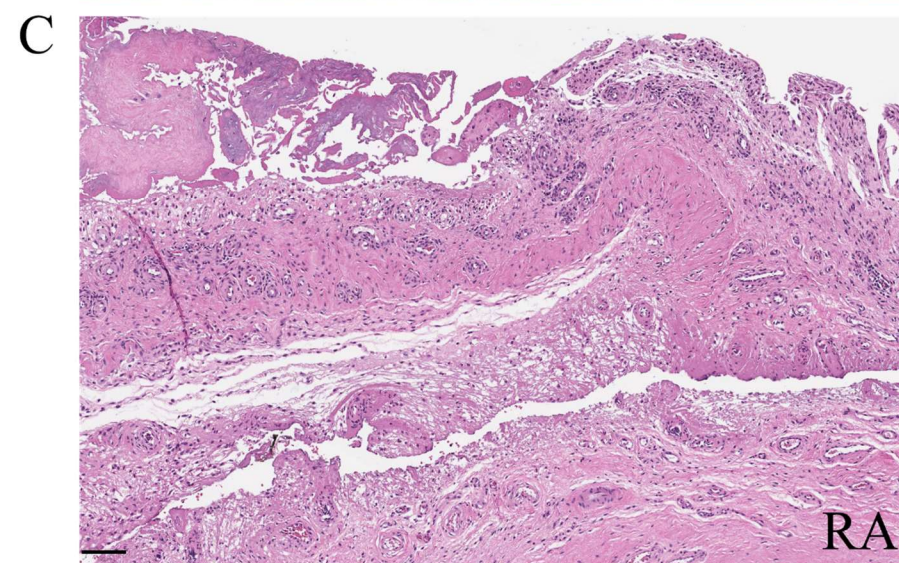

Supplement: Supplementary file 1 — Supplementary Figure S1. The Krennʼs synovitis scores (mean + SE) in the synovial tissues of osteoarthritis (OA) and rheumatoid arthritis (RA) patients (n = 6/group) (panel A) and representative images of hematoxylin–eosin-stained histological sections of OA and RA synovial tissues (panels B–C), scalebar 100 μm. There were no significant differences in the synovitis scores between the diagnoses. In panel B, OA synovial tissue shows slight hyperplasia, moderate stromal activation with increased vasculature, and moderate inflammatory infiltration. In panel C, RA synovial tissue shows superficial necrosis of the lining layer and thickening of the synovial stroma (PDF 664 KB) [file 10753_2023_1816_MOESM1_ESM.pdf]

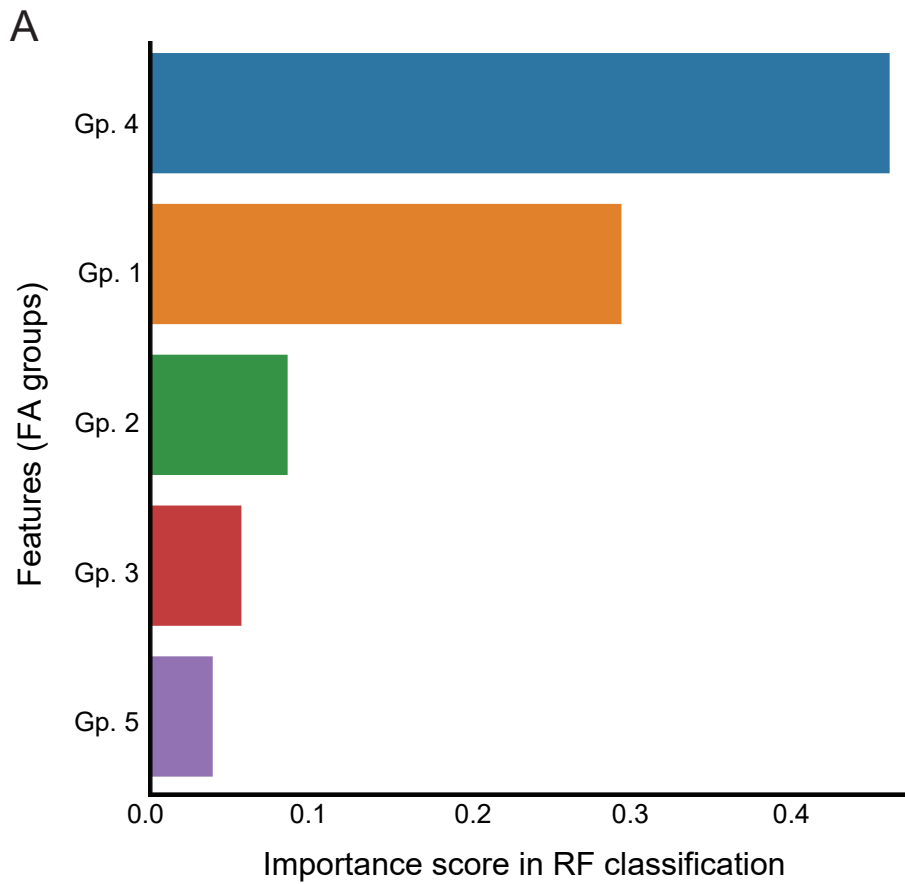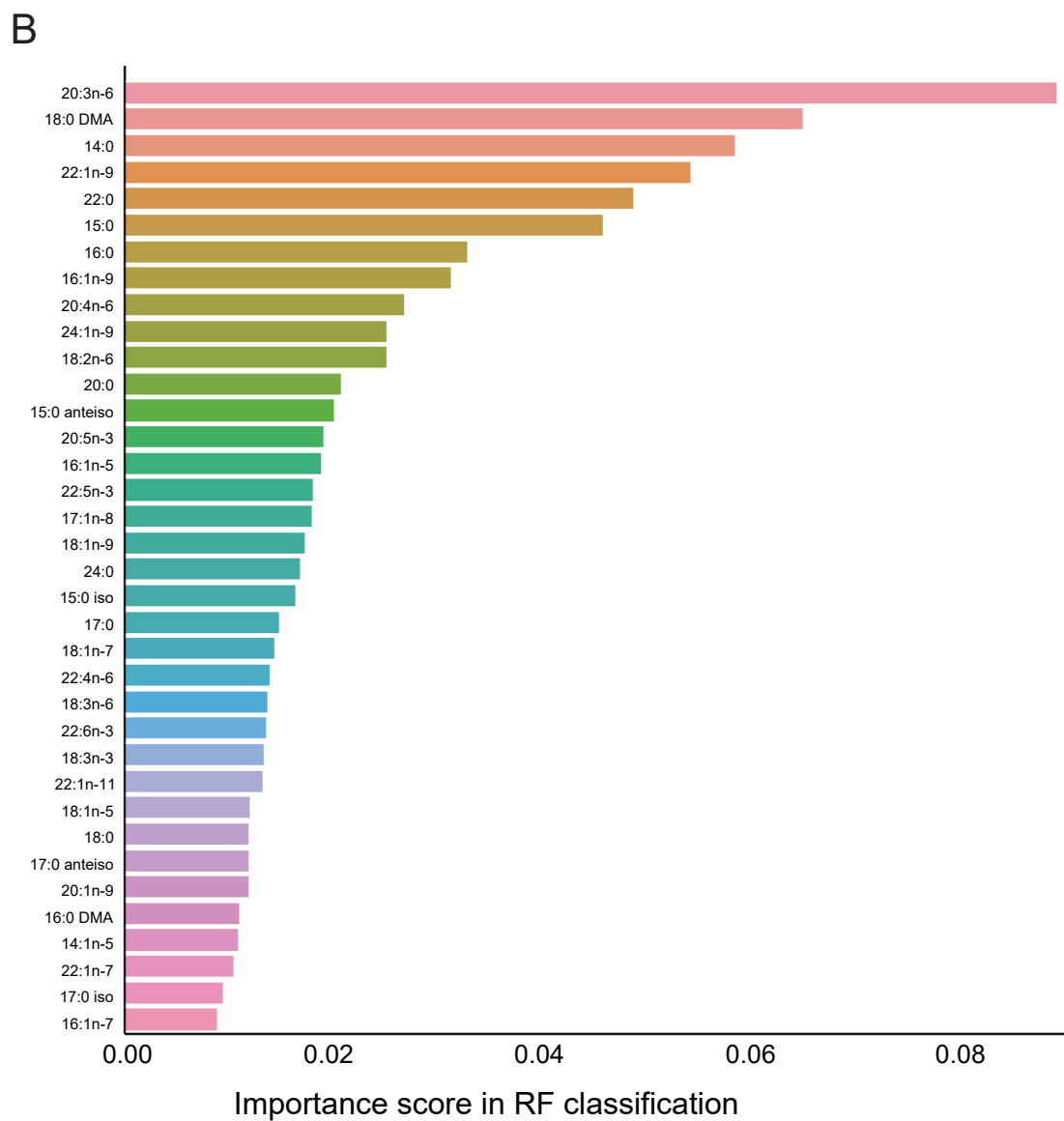

Supplement: Supplementary file 2 — Supplementary Figure S2. The variables 20:3n-6 and saturated fatty acids (FAs) prevail in Random Forest (RF)-based classification of synovium samples based on individual FAs only, excluding FA sums and derived ratios. In panel A, the bar chart shows the feature importance score of each FA group (Gp.) in the RF-based classification of the diagnosis of the samples (see Methods). FA groups were obtained from the groups shown in Fig. 3, where sums and derived ratios were removed. The presented scores are averages of the FA group importance scores over 100 RFs of 100 trees each, run on a 1000-fold enriched dataset. In panel B, the bar chart shows the feature importance score for each individual FA in the RF-based classification of the diagnosis of the samples. The scores presented are averages of the FA importance scores over 100 RFs of 100 trees each (PDF 136 KB) [file 10753_2023_1816_MOESM2_ESM.pdf]

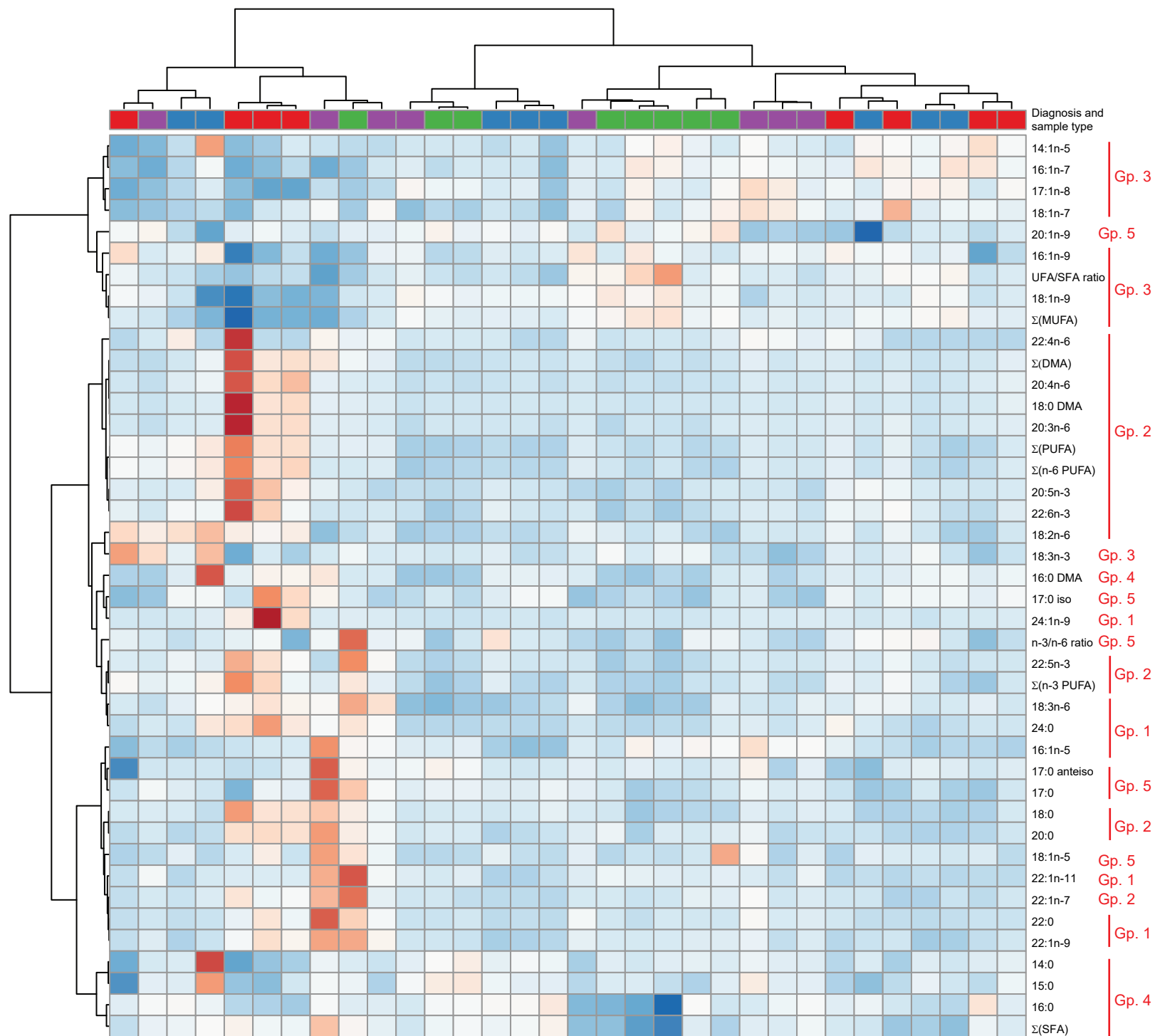

Supplement: Supplementary file 3 — Supplementary Figure S3. Fatty acid (FA) groups are also identifiable across tissues and bear minimal tissue-dependence. Hierarchical clustering clustergram showing the FA Z-scores (rows) in samples (columns) as determined by gas chromatography, color-coded as indicated. For the bonds on the left of the clustergram, the distance of bonds to color-coded clustergram increases with the dissimilarity between FAs across the samples. For the bonds at the top of the clustergram, the distance of bonds to color-coded clustergram increases with the dissimilarity between the samples across the FA space. The clustering was performed with ClustVis using the Ward method [25]. The diagnosis and the tissue of origin of the corresponding sample (RA = rheumatoid arthritis; OA = osteoarthritis; IFP = infrapatellar fat pad) are indicated on top of the clustergram. FA groups (Gp.), identified in Fig. 3, are indicated with red vertical bars on the right side of the image (PDF 220 KB) [file 10753_2023_1816_MOESM3_ESM.pdf]

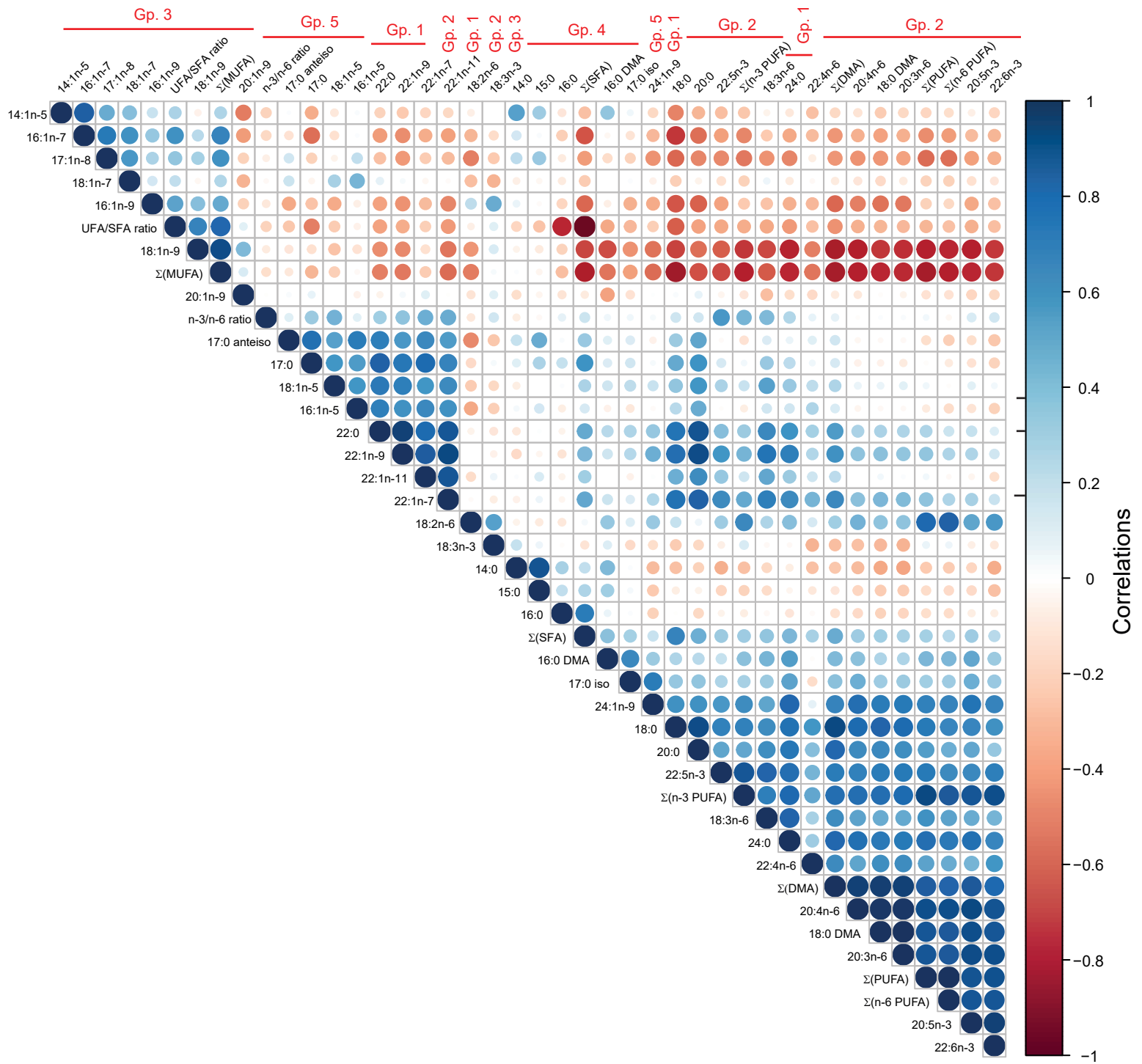

Supplement: Supplementary file 4 — Supplementary Figure S4. Fatty acid (FA) proportions are well correlated across diagnoses and tissues within FA groups. Correlogram showing the Pearson correlation coefficients between all pairs of FAs, FA sums, and derived ratios across all samples (RA and OA, synovial membranes and infrapatellar fat pads). Dark color (blue or red) indicates strong positive (respectively negative) correlations in the FA levels, irrespective of diagnosis. The correlogram was plotted using the Corrplot function in R. The FA groups (Gp.), identified in Fig. 3, are indicated on the top of the correlogram. (PDF 409 KB) [file 10753_2023_1816_MOESM4_ESM.pdf]
