# Supplementary material for: Increased n-6 Polyunsaturated Fatty Acids Indicate Pro- and Anti-Inflammatory Lipid Modifications in Synovial Membranes with Rheumatoid Arthritis
Source: Inflammation. 2023 May 4;46(4):1396–413. doi: 10.1007/s10753-023-01816-3 (PMC10359413; doi:10.1007/s10753-023-01816-3)
Supplement: Supplementary file 5 — Supplementary Material S1. Random Forest analysis code in Python as an annotated Jupyter notebook (Mustonen_et_al_RF_analysis.ipynb) together with the fatty acid level Z-scores for synovial membranes (Synovial_withZscores.xlsx) provided as a single.zip archive (Mustonen_et_al_RF_analysis.zip). (ZIP 228 KB) [file 10753_2023_1816_MOESM5_ESM.zip › input/Z scores synovium.pdf]

no	Diagnosis	Zf140	Zf141n5	Zf150iso	Zf150anteiso	Zf150	Zf160DMA	Zf160	Zf161n9
2	1	-0.46015	-0.49929	-0.88579	-0.3356	-0.4769	-0.45288	-0.11268	1.10212
3	1	-1.21174	-1.07806	1.16533	-0.51796	-0.75571	-0.20729	-1.11985	-2.11283
5	2	-0.01731	0.73082	-0.26841	-0.67181	-0.84899	-0.69124	0.24421	0.51548
6	2	0.24997	-0.64066	0.15263	-0.08431	0.62242	-0.19644	0.8871	0.07793
7	2	-0.01567	-0.54016	-0.67451	-0.1129	0.23746	-0.41389	0.91648	0.2709
8	1	-0.74843	-0.8872	-0.17672	0.37436	-0.89107	0.63967	-1.1334	-1.10896
10	2	3.16646	2.30039	2.40608	3.04338	2.90097	3.33784	0.31207	1.2784
13	2	0.60973	-0.34887	-0.02063	0.01937	0.34849	-0.22879	0.95475	-0.32727
15	2	0.10858	0.28273	0.64738	0.52144	0.48045	-0.01876	-0.99576	-0.02368
17	2	-0.24663	0.73016	0.26022	-0.11963	-0.17369	0.06384	-1.03902	0.35051
18	1	-0.59611	0.60314	-0.38515	-0.50163	-0.36538	-0.39027	-0.65106	0.6619
19	1	-1.11299	-1.34934	-2.20716	-1.88151	-1.62439	-1.29549	-0.0647	1.71767
20	1	0.0889	1.41765	0.16966	-0.36298	-0.37114	-0.44434	1.73827	-1.43477
21	2	0.45648	-0.99433	0.06212	-0.279	0.36631	-0.28043	1.51264	-0.0383
22	1	0.29965	0.53546	0.55454	0.30475	0.62832	-0.01333	-0.05577	-0.49013
23	1	-0.57075	-0.26244	-0.79958	0.60402	-0.07713	0.5918	-1.39329	-0.43898

Zf161n7	Zf161n5	Zf170iso	Zf170anteiso	Zf170	Zf171n8	Zf180DMA	Zf180	Zf181n9	Zf181n7
0.1243	2.37814	-0.15554	-0.83873	-0.23968	0.74796	-0.18074	-0.20235	0.32298	0.05222
-1.44402	0.44998	-1.04782	-0.11529	-1.8174	-0.9545	3.10375	2.65227	-2.19319	-1.18561
1.39123	0.55767	-0.78168	-1.23106	-1.17095	0.2141	-0.53845	-0.76552	0.22335	0.54932
-0.35297	-0.54724	-0.53568	0.27178	0.80769	0.00427	-0.5645	-0.3434	0.76001	-0.29099
-0.65641	-0.49663	0.06864	0.02903	0.74311	-0.48466	-0.428	-0.19177	-0.15132	-0.65392
-1.15486	1.03492	2.69837	-0.09528	1.49564	-1.40794	1.0428	1.45818	-0.88324	-0.28608
0.55602	0.13106	0.08127	0.05145	-0.08144	0.15871	-0.25716	-0.55542	-1.7106	-0.50104
-0.40979	-1.3532	0.1917	0.08535	0.71587	0.35482	-0.49477	-0.22217	0.78538	-0.48722
0.4313	-0.57205	-0.00902	1.17443	0.21764	1.42569	-0.58664	-0.68947	1.17642	0.20185
1.50813	-0.02952	-0.02098	0.83234	-0.87425	1.36035	-0.51388	-0.80806	0.89588	0.63398
1.05314	0.18546	0.0342	0.68219	-0.87262	1.19575	-0.37541	-0.751	0.06777	2.94372
-1.11645	-1.50845	-1.8133	-2.93045	-0.20352	-1.29378	-0.4808	-0.26724	0.94475	-1.14328
1.35686	-0.18252	-0.38148	0.04575	-1.22482	-0.10933	-0.43787	-0.75033	-0.20134	0.67226
-1.15348	-1.0081	0.05151	0.35241	1.496	-0.86595	-0.53515	0.20316	0.85993	-1.06943
0.48172	-0.29972	0.13879	0.81723	0.03194	1.11313	-0.05045	-0.26983	0.20208	0.41861
-0.61472	1.26021	1.48103	0.86884	0.97678	-1.45863	1.29729	1.50294	-1.09887	0.14562

Zf181n5	Zf182n6	Zf183n6	Zf183n3	Zf200	Zf201n9	Zf203n6	Zf204n6	Zf205n3	Zf220
-1.06564	0.03664	-0.23779	0.08139	-0.31706	-0.74596	-0.22934	-0.29812	-0.15943	-0.1451
0.11093	0.84512	1.32285	-1.32686	1.97909	0.39786	3.10212	2.72184	2.74097	1.06572
0.80599	-0.76729	0.39302	-0.3934	-0.32164	-2.66329	-0.51651	-0.52859	0.10907	-0.44663
0.83697	-0.82376	-1.52266	0.07407	-0.64934	1.05318	-0.55815	-0.70252	-0.47124	-0.55109
0.77729	1.2558	0.07711	0.21434	-0.5123	-0.33241	-0.38039	-0.25078	-0.59355	-0.44465
2.79401	0.57309	2.01515	-0.15849	2.11408	0.90371	1.09932	1.26369	1.66003	3.11343
-0.9119	1.84474	0.97138	2.06258	-0.39142	-1.39418	-0.26464	0.04442	0.69442	-0.27799
-0.58386	-0.96846	-1.26697	-0.54655	-0.50101	0.49982	-0.5681	-0.59847	-0.7434	-0.45643
0.01047	-0.68618	-0.66428	0.52705	-0.61195	0.1587	-0.64514	-0.69961	-0.5911	-0.55659
-0.18049	-1.05207	-0.53007	-0.44606	-0.60435	0.08756	-0.5215	-0.61024	-0.70375	-0.45527
0.23914	0.02871	0.39209	0.08177	-0.55071	-0.33572	-0.05949	-0.26481	-0.02862	-0.44014
-1.09247	1.47976	-0.57566	2.46485	-0.46263	1.00117	-0.50071	-0.59864	-0.37262	-0.47142
0.35234	-1.14447	0.08327	-0.9428	-0.53681	-0.34926	-0.34559	-0.31311	-0.77486	-0.35888
-0.77337	-0.81085	-1.00196	-0.5018	-0.31107	1.25931	-0.68204	-0.73437	-0.84597	-0.54198
-0.80597	-0.61857	-0.66007	-0.45227	-0.22757	0.20847	-0.13585	-0.09592	-0.58495	-0.33506
-0.51346	0.80779	1.20459	-0.73782	1.90468	0.25105	1.20602	1.66522	0.66503	1.30208

Zf2221n11	Zf221n9	Zf221n7	Zf224n6	Zf225n3	Zf240	Zf226n3	Zf241n9	ZSUMSAT	ZSUMMUFA
-0.74348	-0.53019	-0.2339	0.57464	-0.14939	0.75297	0.01323	-0.21738	-0.4728	0.29404
1.84152	1.22966	2.80607	3.3238	2.77727	1.43684	3.00849	0.80324	1.43146	-2.46548
-0.11944	-0.21577	-0.23842	0.22383	-0.46968	-0.32931	-0.22404	-0.46672	-0.69714	0.78018
-0.99286	-0.69849	-0.57704	-0.15118	-0.23941	-0.67523	-0.1871	-0.54558	0.35033	0.41183
-0.76707	-0.5961	-0.38866	1.09975	0.18953	-0.56356	0.15043	-0.38338	0.41743	-0.46714
1.60572	2.7096	1.09545	-0.42255	1.73874	2.36829	1.44833	3.2234	0.60681	-1.12269
1.1972	-0.22086	0.38392	-0.45091	-0.25765	1.1328	0.0196	-0.1659	1.02577	-1.04675
-0.87544	-0.59272	-0.60832	-0.47259	-0.69204	-0.71229	-0.74154	-0.48635	0.65652	0.38234
-0.12262	-0.77722	-0.79428	-0.47283	-0.61231	-0.77218	-0.55003	-0.56625	-1.34885	1.12474
-0.74183	-0.68869	-0.29262	-0.46917	-0.85224	-0.63811	-0.92579	-0.41353	-1.6729	1.4027
-0.71843	-0.1815	-0.76422	-0.46863	0.31623	-0.59915	0.33454	-0.30276	-1.49051	0.70929
-0.12572	-0.28877	-0.50528	-0.4599	-0.40532	-0.68297	-0.19048	-0.48817	-0.9093	0.16496
-0.53558	-0.33076	-0.61471	-0.46471	-0.76357	-0.51893	-0.99076	-0.42058	0.42588	0.45776
-0.11313	-0.51378	-0.65902	-0.46948	-0.72489	-0.77082	-0.85108	-0.5641	1.41761	0.07628
-0.52562	-0.03805	-0.16395	-0.46635	-0.59755	-0.49885	-0.41187	-0.27106	-0.18782	0.39445
1.73678	1.73363	1.55499	-0.45374	0.7423	1.0705	0.09806	1.26512	0.44751	-1.09649

ZSUMPUFA	ZSUMn6PUFA	ZSUMn3PUFA	ZSUMDMA	ZUNSATSAT	Zn3n6
-0.11844	-0.13378	-0.03444	-0.22855	0.37256	0.15787
2.44228	2.37178	2.55999	3.016	-1.35597	0.22158
-0.65112	-0.69696	-0.37335	-0.606	0.62609	0.87157
-0.75887	-0.85562	-0.22791	-0.57519	-0.39229	1.98739
0.45134	0.51262	0.11768	-0.46626	-0.45407	-0.74713
1.16006	1.05608	1.55311	1.09405	-0.66971	0.73929
0.86975	0.88942	0.69747	0.12805	-0.96389	-0.3269
-0.90646	-0.8866	-0.91997	-0.51059	-0.65173	-0.27106
-0.75292	-0.80792	-0.42222	-0.57665	1.39208	1.13109
-0.96116	-0.92756	-1.03553	-0.496	1.81823	-0.54772
-0.08453	-0.1649	0.30744	-0.41207	1.56806	1.03521
0.35911	0.32423	0.49369	-0.61831	0.86341	0.2821
-0.87741	-0.79014	-1.21596	-0.47939	-0.46079	-1.53091
-0.9247	-0.89262	-0.99505	-0.55601	-1.22572	-0.51348
-0.44764	-0.3977	-0.64623	-0.05092	0.08039	-0.74633
1.20071	1.39966	0.14129	1.33783	-0.54666	-1.74257
