# Supplementary material for: Increased n-6 Polyunsaturated Fatty Acids Indicate Pro- and Anti-Inflammatory Lipid Modifications in Synovial Membranes with Rheumatoid Arthritis
Source: Inflammation. 2023 May 4;46(4):1396–413. doi: 10.1007/s10753-023-01816-3 (PMC10359413; doi:10.1007/s10753-023-01816-3)
Supplement: Supplementary file 6 — Supplementary Table S1. Grouping of fatty acids, dimethyl acetals (DMAs, derivatives of plasmalogen phospholipid alkenyl chains), their sums, and derived ratios based on hierarchical clustering (PDF 411 KB) [file 10753_2023_1816_MOESM6_ESM.pdf]

Supplementary Table S1. Grouping of fatty acids, dimethyl acetals (DMAs, derivatives of plasmalogen phospholipid alkenyl chains), their sums, and derived ratios based on hierarchical clustering. Group 4 consists of 2 subgroups that were fused for biological reasons (short saturated fatty acids).

Group 1

16:1n-5	CHEBI:35464
22:1n-9	CHEBI:28792
22:0	CHEBI:28941
24:1n-9	CHEBI:44247
22:1n-11	CHEBI:32428
18:3n-6	CHEBI:28661
24:0	CHEBI:28866

Group 2

22:1n-7	CHEBI:180035
20:4n-6	CHEBI:15843
Sum DMA	
DMA 18:0	
20:3n-6	CHEBI:53486
18:0	CHEBI:28842
20:0	CHEBI:28822
18:2n-6	CHEBI:17351
22:4n-6	CHEBI:53487
Sum PUFA	
Sum n-6 PUFA	
20:5n-3	CHEBI:28364
Sum n-3 PUFA	
22:5n-3	CHEBI:53488
22:6n-3	CHEBI:28125

Group 3

16:1n-9	CHEBI:35465
18:3n-3	CHEBI:27432
18:1n-9	CHEBI:16196
Sum MUFA	
14:1n-5	CHEBI:27781
16:1n-7	CHEBI:28716
18:1n-7	CHEBI:50464
17:1n-8	CHEBI:84328
UFA/SFA ratio	

Group 4

15:0 iso	CHEBI:39250
15:0 anteiso	CHEBI:39251
DMA 16:0	
14:0	CHEBI:28875
15:0	CHEBI:42504
16:0	CHEBI:15756
Sum SFA	

Group 5

18:1n-5	CHEBI:82617
n-3/n-6 PUFA ratio	
20:1n-9	CHEBI:32425
17:0 anteiso	CHEBI:84874
17:0 iso	CHEBI:70850
17:0	CHEBI:32365

ChEBI (Chemical Entities of Biological Interest) IDs from https://www.ebi.ac.uk/chebi/
