# Supplementary material for: Increased n-6 Polyunsaturated Fatty Acids Indicate Pro- and Anti-Inflammatory Lipid Modifications in Synovial Membranes with Rheumatoid Arthritis
Source: Inflammation. 2023 May 4;46(4):1396–413. doi: 10.1007/s10753-023-01816-3 (PMC10359413; doi:10.1007/s10753-023-01816-3)
Supplement: Supplementary file 7 — Supplementary Table S2. Proportions (mol-%) of fatty acids and alkenyl chains (detected as dimethyl acetal derivatives DMAs) and their sums and ratios in the synovium of rheumatoid arthritis (RA) and osteoarthritis (OA) patients (mean ± SE) (PDF 314 KB) [file 10753_2023_1816_MOESM7_ESM.pdf]

Supplementary Table S2. Proportions (mol-%) of fatty acids and alkenyl chains (detected as dimethyl acetal derivatives DMAs), listed in the order of retention times of chromatographic peaks, and their sums and ratios in the synovium of rheumatoid arthritis (RA) and osteoarthritis (OA) patients (mean ± SE).

**Fatty acid**	**RA**	**OA**	P^{a}
14:0	2.450 ± 0.257	3.942 ± 0.536	0.010
14:1n-5	0.589 ± 0.147	0.753 ± 0.163	0.505
15:0*i*	0.045 ± 0.007	0.058 ± 0.006	0.279
15:0*ai*	0.071 ± 0.010	0.093 ± 0.016	0.382
15:0	0.232 ± 0.024	0.335 ± 0.040	0.050
DMA 16:0	0.070 ± 0.011	0.090 ± 0.023	0.721
16:0	23.925 ± 0.892	25.690 ± 0.833	0.105
16:1n-9	0.603 ± 0.088	0.702 ± 0.032	0.328
16:1n-7	7.057 ± 1.328	8.209 ± 1.199	0.442
16:1n-5	0.094 ± 0.015	0.065 ± 0.008	0.105
17:0*i*	0.123 ± 0.023	0.112 ± 0.006	0.959
17:0*ai*	0.160 ± 0.015	0.173 ± 0.008	0.442
17:0	0.146 ± 0.023	0.173 ± 0.019	0.382
17:1n-8	0.255 ± 0.029	0.293 ± 0.020	0.234
DMA 18:0	0.480 ± 0.194	0.052 ± 0.016	0.003
18:0	6.399 ± 1.618	3.363 ± 0.443	0.234
18:1n-9	35.566 ± 2.385	40.399 ± 2.249	0.130
18:1n-7	3.196 ± 0.248	2.977 ± 0.116	0.645
18:1n-5	0.155 ± 0.034	0.155 ± 0.020	0.645
18:2n-6	9.886 ± 0.747	8.640 ± 0.990	0.279
18:3n-6	0.062 ± 0.007	0.043 ± 0.007	0.083
18:3n-3	0.790 ± 0.137	0.873 ± 0.103	0.442
20:0	0.099 ± 0.025	0.043 ± 0.003	0.065
20:1n-9	0.427 ± 0.030	0.381 ± 0.064	0.959
20:3n-6	0.542 ± 0.160	0.162 ± 0.018	0.001
20:4n-6	3.733 ± 1.220	0.835 ± 0.273	0.028
20:5n-3	0.286 ± 0.071	0.157 ± 0.031	0.130
22:0	0.108 ± 0.045	0.015 ± 0.003	0.010
22:1n-11	0.007 ± 0.002	0.005 ± 0.001	0.279
22:1n-9	0.056 ± 0.014	0.019 ± 0.003	0.007
22:1n-7	0.009 ± 0.003	0.004 ± 0.001	0.195
22:4n-6	0.184 ± 0.139	0.098 ± 0.059	0.442
22:5n-3	0.686 ± 0.134	0.407 ± 0.037	0.083
24:0	0.154 ± 0.051	0.050 ± 0.028	0.065
22:6n-3	1.107 ± 0.274	0.598 ± 0.089	0.161
24:1n-9	0.247 ± 0.108	0.035 ± 0.011	0.028
Σ SFA	33.912 ± 1.200	34.048 ± 1.448	1.000
Σ MUFA	48.259 ± 3.382	53.996 ± 2.466	0.234
Σ PUFA	17.278 ± 2.304	11.814 ± 1.499	0.050
Σ n-6 PUFA	14.408 ± 1.904	9.778 ± 1.297	0.028

Σ n-3 PUFA	2.870 ± 0.446	2.036 ± 0.225	0.161
Σ DMA	0.550 ± 0.200	0.142 ± 0.038	0.050
UFA/SFA	1.960 ± 0.112	1.973 ± 0.134	0.959
n-3/n-6 PUFA	0.197 ± 0.015	0.213 ± 0.014	0.645
Prod/pred n-6 PUFA	0.415 ± 0.115	0. 104 ± 0.018	0.007
Prod/prec n-3 PUFA	2.333 ± 0.948	0.869 ± 0.103	0.105
Δ9-desaturation index	1.443 ± 0.141	1.615 ± 0.142	0.721
Δ6-desaturation index	0.006 ± 0.001	0.005 ± 0.001	0.130
Δ5-desaturation index	6.205 ± 0.602	4.468 ± 0.956	0.130
DBI	0.994 ± 0.056	0.847 ± 0.025	0.028
TACL	17.438 ± 0.105	17.178 ± 0.030	0.028

i = *iso*-methyl-branch, *ai* = *anteiso*-methyl-branch, SFA = saturated fatty acid, MUFA = monounsaturated fatty acid, PUFA = polyunsaturated fatty acid, UFA = unsaturated fatty acid (MUFA + PUFA), prod = product, prec = precursor, DBI = double bond index, TACL = total average chain length, [a]Mann–Whitney U test
