# Supplementary material for: Increased n-6 Polyunsaturated Fatty Acids Indicate Pro- and Anti-Inflammatory Lipid Modifications in Synovial Membranes with Rheumatoid Arthritis
Source: Inflammation. 2023 May 4;46(4):1396–413. doi: 10.1007/s10753-023-01816-3 (PMC10359413; doi:10.1007/s10753-023-01816-3)
Supplement: Supplementary file 8 — Supplementary Table S3. Medical data of the patients with rheumatoid arthritis (PDF 180 KB) [file 10753_2023_1816_MOESM8_ESM.pdf]

Supplementary Table S3. Medical data of the patients with rheumatoid arthritis.

ID	Diagnosis year	Post-traumatic osteoarthritis	Anti-rheumatic medication	Glucocorticoids	Biological drugs	Analgesics
1	1993		hydroxychloroquine, podophyllotoxin	methylprednisolone		P.R.N.
2	1972		none for several months	methylprednisolone		P.R.N.
3	2005		salazosulfapyridine, leflunomide			P.R.N.
4	2016		methotrexate, folic acid	methylprednisolone		P.R.N.
5	2001		leflunomide	methylprednisolone		P.R.N.
6	1983		salazosulfapyridine, hydroxychloroquine			paracetamol P.R.N.
7	2007		salazosulfapyridine	methylprednisolone		
8	1996	Open arthrotomy with debridement during 1970’s	methotrexate, folic acid	methylprednisolone	adalimumab	paracetamol P.R.N.

P.R.N. = pro re nata
